# Preparation of dendritic carboranyl glycoconjugates as potential anticancer therapeutics†

**DOI:** 10.1039/d0ra07264h

**Published:** 2020-09-18

**Authors:** Biswa Ranjan Swain, Chandra Sekhara Mahanta, Bibhuti Bhusan Jena, Swaraj Kumar Beriha, Bismita Nayak, Rashmirekha Satapathy, Barada P. Dash

**Affiliations:** Department of Chemistry, Ravenshaw University Cuttack Odisha 753 003 India rashmi.satapathy@gmail.com rashmi@ravenshawuniversity.ac.in; Department of Chemistry, Siksha ‘O’ Anusandhan (Deemed to be University) Bhubaneswar Odisha 751030 India barada.dash@gmail.com baradadash@soa.ac.in; Department of Life Science, National Institute of Technology Rourkela Odisha 769 008 India

## Abstract

A series of carborane-appended glycoconjugates containing three and six glucose and galactose moieties have been synthesized *via* Cu(i)-catalyzed azide–alkyne [3 + 2] click cycloaddition reaction. The carboranyl glycoconjugates containing three glucose and galactose moieties were found to be partially water soluble whereas increasing the number to six made them completely water soluble. The evaluation of cytotoxicities and IC_50_ values of newly synthesized carboranyl glycoconjugates was carried out using two cancerous cell lines (MCF-7 breast cancer cells and A431 skin cancer cells) and one normal cell line (HaCaT skin epidermal cell line). All carboranyl glycoconjugates showed higher cytotoxicities towards cancerous cell lines than the normal cell line. Carboranyl glycoconjugates containing three glucose and galactose moieties (compounds 15 and 17) were found to be more cytotoxic than the glycoconjugates containing six glucose and galactose moieties (compounds 19 and 21). Moreover, administration of 100 μM concentrations of compounds 15 and 17 inhibited up to 83% of MCF-7 breast cancer cells and up to 79% A431 skin cancer cells. However, administration of similar concentrations of carboranyl glycoconjugates could inhibit only up to 35–45% of HaCaT normal epidermal cells. Thus, due to the higher cytotoxicities of dendritic carboranyl glycoconjugates towards cancer cells over healthy cells, they could potentially be useful for bimodal treatment of cancer such as chemotherapy agents and boron neutron capture therapy (BNCT) agents as well.

## Abstract 2

*o*-Carborane-appended glycoconjugates containing multiple glucose and galactose moieties are found to be more cytotoxic towards cancer cells than normal cells.

## Graphical abstract

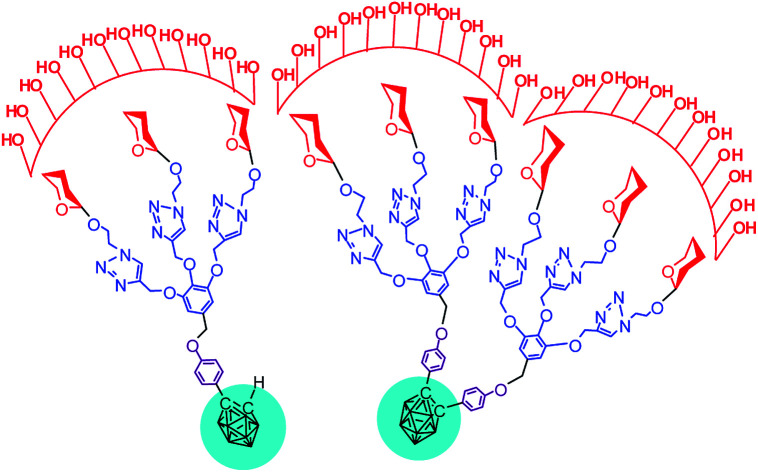

## Introduction

Boron-rich icosahedral carboranes have been used for several medicinal applications. The most promising among them is their use in the treatment of cancer.^1–4^ Treatment of cancer can be accomplished *via* several approaches such as chemotherapy, radiation therapy, surgery, laser therapy, alternative medicines and immunotherapy. However, none of these methods are fully effective because of inherent limitations associated with their use. The commonly used chemotherapy approach for the treatment of cancer limits its effectiveness due to the toxicity associated with the chemotherapy agents towards healthy cells in the body. Traditionally icosahedral carboranes have been used to synthesize boron delivery platforms for the treatment of cancer *via* boron neutron capture therapy (BNCT).^5,6^ For an effective boron neutron capture therapy, the required number of ^10^B atoms has been estimated to be 10^9^ per tumor tissue, which is approximately 35 μg boron per g of tumor tissue. But the surrounding healthy cells must not contain more than 5 μg boron per g of tumor tissue to avoid the damage caused by the radiation to normal healthy cells.^7^

In addition to their use for BNCT applications, derivatives of icosahedral carboranes have also been found to show cytotoxicity towards cancer cells. Recently dendritic molecules containing multiple *o*-carborane clusters have been found to show significantly higher cytotoxicity towards MCF-7 breast cancer cell lines than the commonly used chemotherapy agent cisplatin.^8^ Derivatives of icosahedral carboranes have also shown cytotoxicity towards glioma cells.^9,10^ Therefore, derivatives of carborane could be suitable as chemotherapy agents for treatment of cancer. Carboranes are very favorably used for boron delivery because of their high boron content and stability to catabolism. However, carboranes possess hydrophobicity and their low water solubility often limits their use in medicinal applications. Moreover, derivatives of carboranes need to be delivered selectively into the tumor tissues in adequate amounts for the successful cancer therapy. Dendrimers and dendritic macromolecules often find applications as drug delivery platforms. Because of the dendrimer's unique branching architecture and high number of surface functional groups; it can carry large payloads of therapeutic molecules. Cellular uptake of dendrimers-based drug delivery systems has been proved to be significantly higher.^11–13^ Due to the leaky vasculature of tumor tissues, macromolecular and dendrimers-based drug delivery systems are preferentially transported to the tumor tissues and accumulate in them in a process known as the enhanced permeability and retention (EPR) effect.^14,15^ Macromolecular and dendritic drug delivery agents are also found to be superior in terms of accumulation and retention in the tumor tissues. The biological evaluation of branched dendrimer containing nine carborane cages was carried out using human liver cancer cells (SK-Hep1). The highest boron accumulation up to 2540 ng of boron/5 × 10^5^ cells was observed at 50 μM concentration of the dendrimer over a period of 20 hours. The high accumulation of the dendrimer into the tumor cells indicates that such dendritic boron drug delivery platforms could be a viable approach for the delivery of boron to the tumor tissues for successful treatment of cancer.^16^

In order to improve the water solubility, carboranes have been linked to some hydrophilic groups, such as hydroxyl groups, water-soluble chains and carbohydrates.^17,18^ It has been found that a minimum of eight hydroxyl groups per carborane is necessary to impart water solubility to the carborane derivatives.^18^ For all living cells, carbohydrates plays vital role as a primary energy source and higher glucose uptake in most of the cancerous cells is a characteristic feature.^19^ The endogenous lectins present on the surface of both normal and malignant cells acts as the specific receptors and messengers for the carbohydrate specific endocytosis of glycoconjugates. However, there is over expression of lectins on the cell surface of cancerous cells. Therefore, carbohydrate–carborane conjugates could be a viable platform for selective delivery of adequate boron into tumor tissues. Glycoconjugates have also been used as specific drug delivery agents in enzyme replacement, immune activation, gene, antiviral, and cancer therapies.^20–22^ Glycoconjugates of polyhedral boron clusters have been synthesized for tumor targeted delivery of boron for successful treatment of cancer.^17,23^ From the earlier reports it has been revealed that hydroxylated glycoconjugates of carboranes showed high accumulation in tumor cells and exhibited high water solubility and low toxicity even at high concentration.^4,24^ Several attempts have been made so far to develop glycoconjugates of carboranes as drugs for successful treatment of cancer *via* boron neutron capture therapy (BNCT).^25–32^ However, a thorough investigation of cytotoxicities of carboranyl glycoconjugates towards cancer cell lines *vis-a-vis* normal cells is necessary to access their efficacy for cancer therapy. In the present work, we describe the synthesis of a series of water-soluble dendritic glycoconjugates of carborane containing three and six glucose and galactose moieties *via* Cu(i)-catalyzed azide–alkyne click cycloaddition reaction. The *in vitro* cytotoxicities of these glycoconjugates have been assessed using two cancer cell lines (MCF-7 breast carcinoma and A431 skin carcinoma) and a normal cell line (HaCaT normal skin epidermal cell) *via* MTT test.

## Result and discussion

The dendritic carboranyl glycosides 15, 17, 19 and 21 containing three and six glucose and galactose moieties were synthesized starting from *p*-iodoanisole following a sequence of reaction conditions and involving Cu(i)-catalyzed azide–alkyne [3 + 2] cycloaddition reaction^16^ followed by transacetylation under Zemplen reaction conditions.^33^ Subsequently, *in vitro* cytotoxicity test was performed in using one normal cell line and two cancerous cell lines.

### Synthesis of *o*-carborane-appended – alkynyl dendrons

**Scheme 1:**
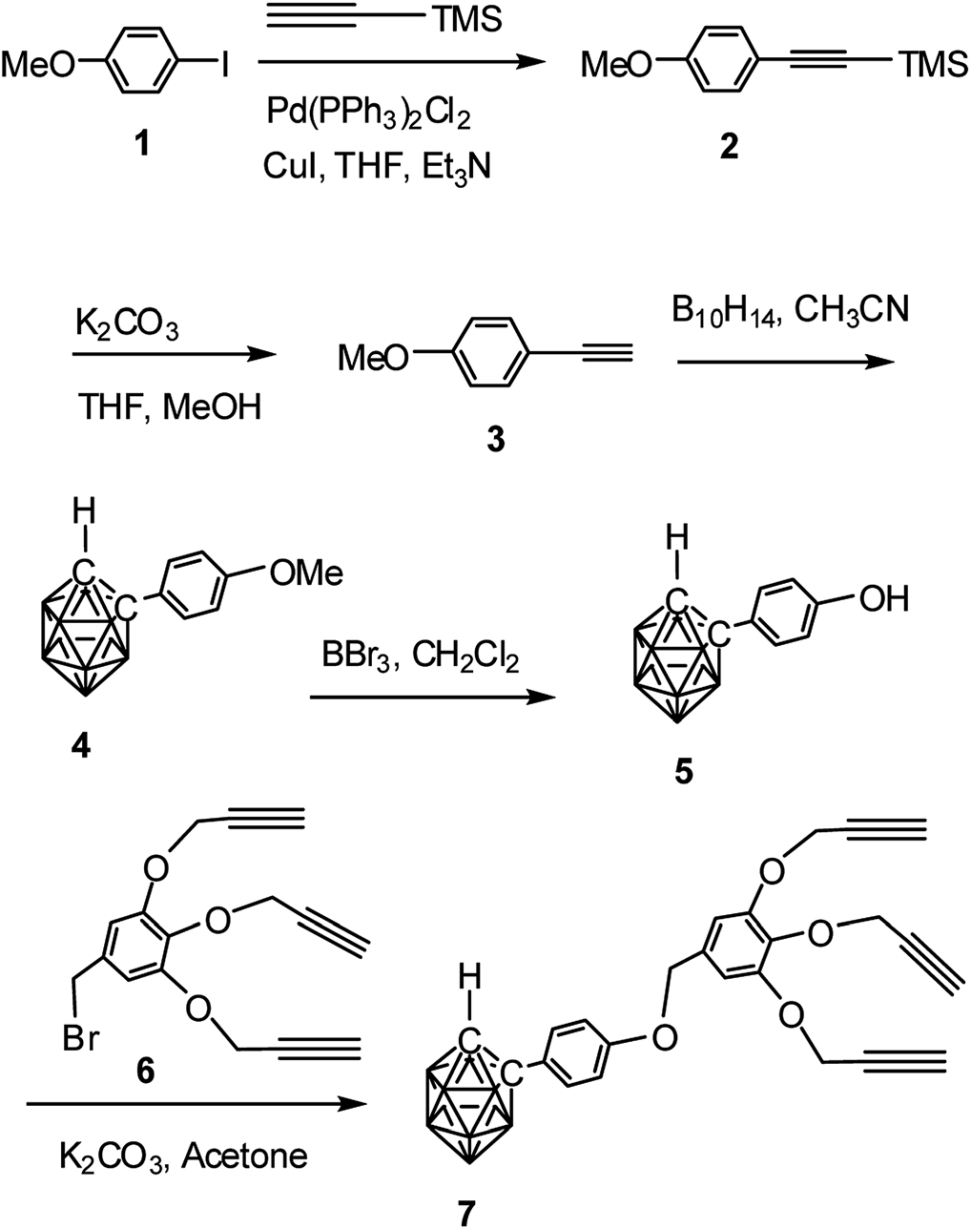
Synthesis of *o*-carborane-appended alkynyl dendron 7 containing three terminal alkyne moieties.

The alkynyl dendron 7 was prepared by a sequence of reaction starting from Sonogashira cross coupling reaction^34–36^ between *p*-iodoanisole (1) and trimethyl silyl acetylene to obtain 2 which upon desilylation using K_2_CO_3_ produced the alkyne 3. Compound 3 was refluxed with decaborane to produce the *o*-carborane derivative 4 in good yield.^37^ The deprotection of methyl ether of compound 4 with BBr_3_ provided the hydroxylated derivative 5.^38^ Compound 4 when refluxed with 6 in acetone/K_2_CO_3_ produced the *o*-carborane-appended alkynyl dendron 7 in 92% yield (Scheme 1). Preparation of compound 6 was accomplished according to a three-steps reaction sequence starting from methyl, 3,4,5-trihydroxy benzoate following literature procedure.^16,39^ The formation of compound 7 has been confirmed by the NMR, mass and IR spectral analysis. Compound 7 shows the characteristic broad singlet (*δ*: 3.81 ppm) for one cage proton and at *δ*: 2.40 ppm for three terminal alkyne protons in the ^1^H NMR spectrum (see ESI†). The ^13^C NMR spectrum also shows characteristics peaks for alkyne carbon and cage carbon.

**Scheme 2:**
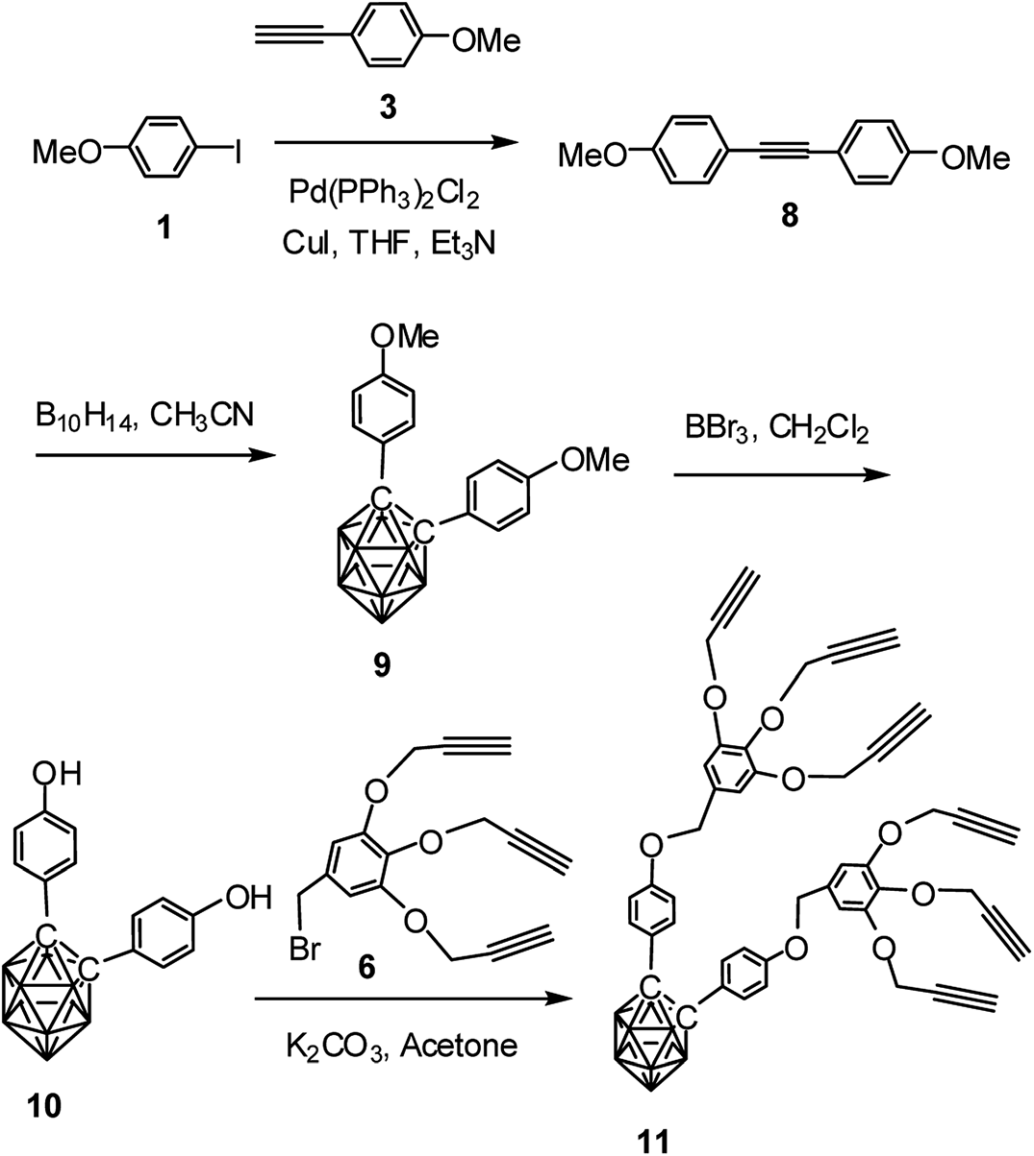
Synthesis of *o*-carborane-appended alkynyl dendron 11 containing six terminal alkyne moieties.

Following similar procedure, alkynyl dendron 11 was prepared when compound 3 underwent Sonogashira cross coupling reaction with 1 followed by treatment with decaborane. The so formed compound 9 upon demethylation with boron tribromide produced the dihydroxy derivative 10. Compound 10 when refluxed with compound 6 in the presence of K_2_CO_3_ in acetone produced the desired *o*-carborane-appended alkynyl dendron with six terminal alkynyl moieties (Scheme 2). The formation of the compound 11 has been confirmed from its NMR, mass and IR spectral data (see ESI†). The compound 11 shows characteristic ^1^H NMR peaks at *δ*: 2.38 ppm and *δ*: 2.35 ppm for the six acetylenic protons and ^13^C NMR peak at *δ*: 85.7 ppm for the two cage carbons. ^11^B NMR confirmed the presence of *o*-carborane cage in the compound. The complete disappearance of the broad peak at 3329 cm^−1^ and appearance of characteristics sharp band at 3280 cm^−1^ represents the alkyne C–H stretching and a band at 2121 represents *ν*(C≡C) in FTIR spectrum for compound 11. The IR spectrum of compound 11 containing *o*-carborane showed strong band at 2589 cm^−1^ corresponding to *ν*(B–H).

### Synthesis of dendritic and water-soluble *o*-carborane–carbohydrate conjugates

**Scheme 3:**
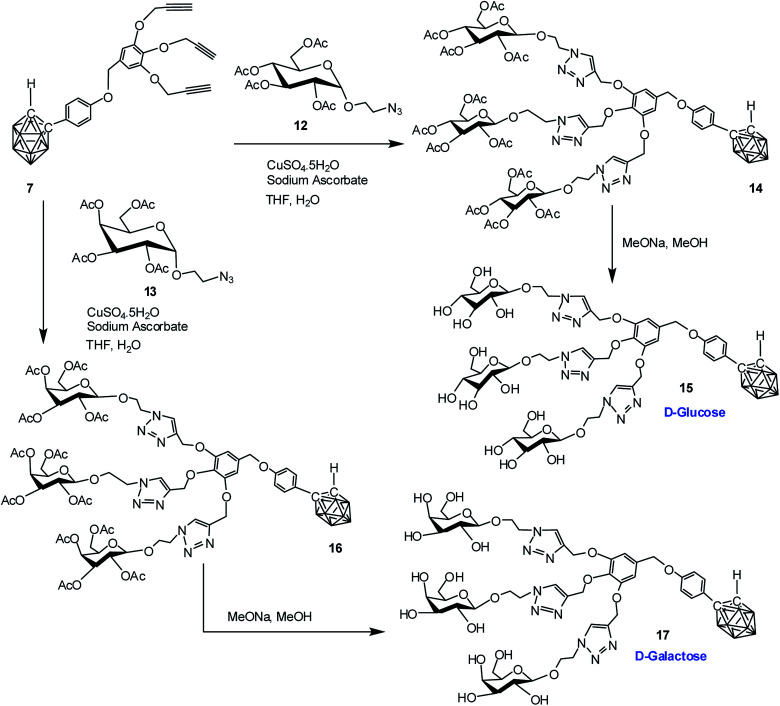
Synthesis of dendritic carboranyl glycoconjugates containing three glucose and galactose moieties.

**Scheme 4:**
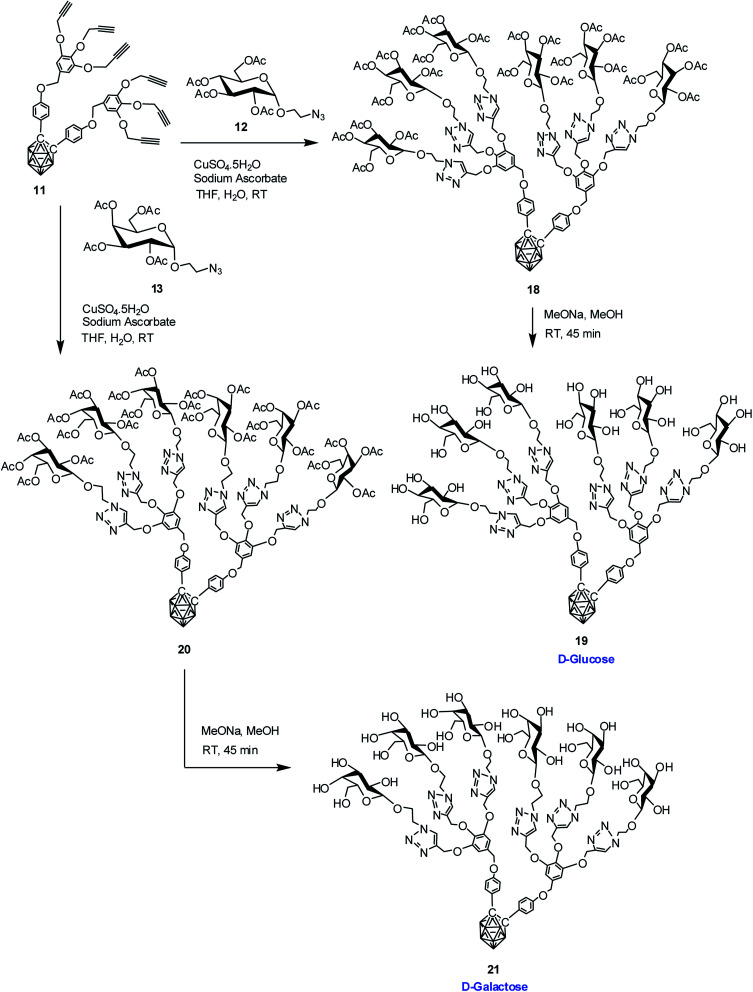
Synthesis of dendritic carboranyl glycoconjugates containing six glucose and galactose moieties.

Commercially available d-glucose and d-galactose were first acetylated in the presence of indium triflate and acetic anhydride as per reported procedure.^40^ Then the sugar azides 12 and 13 were prepared according to the literature procedure.^41,42^ The compound 14 and 16 was obtained in good yield *via* Cu(i)-catalyzed azide–alkyne [3 + 2] cycloaddition reaction^16^ between alkynyl core 7 and glucosyl azide 12 and galactosyl azide 13 respectively. The ^1^H, ^13^C and ^11^B NMR data reveals the formation of the desired compounds. Subsequent de-*O*-acetylation was carried out using sodium methoxide in dry methanol to obtain dendrimer 15 and 17 containing three glucose and galactose moieties respectively. The complete disappearance of the ester protons in ^1^H NMR confirmed the formation of the fully hydroxylated carborane–glucose and carborane–galactose conjugates (Scheme 3). Formation of these products was further confirmed from the mass spectral analysis of the compounds. Following similar approach, reaction of alkynyl core 11 with glucosyl azide 12 and galactosyl azide 13 produced acetylated dendritic compounds 18 and 20 in good yield. The removal of acetyl groups was carried out using sodium methoxide in dry methanol to give water-soluble de-*O*-acetylated dendritic compounds 19 and 21 in good yield (Scheme 4). The complete disappearance of the peak of ester proton in ^1^H NMR and the peaks for carbonyl carbon in ^13^C NMR was observed in spectral analysis indicating the formation of the compound. ^11^B NMR, FTIR and mass spectral analysis confirmed the formation of compounds 19 and 21 (see ESI†). Addition of three glucose and galactose moieties to *o*-carborane clusters (15 and 17) made them partially water soluble and they could be only soluble completely in polar solvents such as methanol and dimethyl sulfoxide. Whereas increasing the number of sugar moieties to six (19 and 21) made them completely water soluble and they were also found to be soluble in polar solvents such as methanol and dimethyl sulfoxide.

### *In vitro* cytotoxicity test

**Fig. 1:**
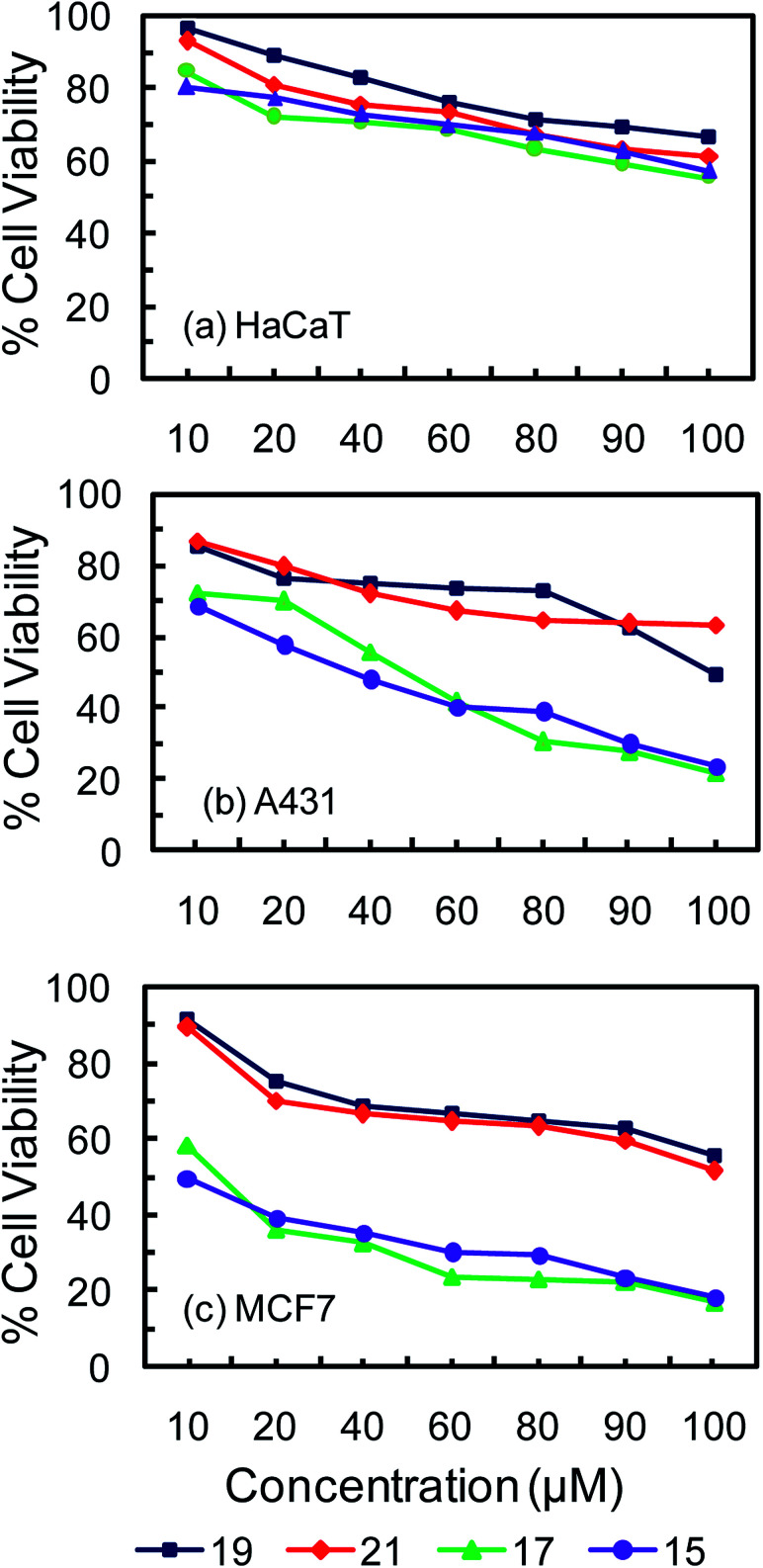
Cytotoxic effect of carboranyl glycoconjugates on different cell lines obtained by MTT assay. (a) HaCaT (normal epidermal cell line), (b) A431 (skin cancer cell line) and (c) MCF-7 (breast cancer cell line).

MTT assay employs a colorimetric method for determining the percentage of viable cells based on mitochondrial dehydrogenase activity measurement at 595 nm. It is considered as an economic, convenient and rapid approach for the detection of viable and dead cells. To test the cytotoxic effect of the glucose and galactose containing carboranyl glycoconjugates, cell viability study was done with the conventional MTT reduction assay.^43,44^*In vitro* cytotoxicity studies of the newly synthesized compounds 15, 17, 19 and 21 were carried out on three different cell lines. Among them two cancer cell lines such as MCF-7 (breast carcinoma) and A431 (skin carcinoma) and one normal cell line, HaCaT (skin epidermal cell) have been used. The results of the *in vitro* cytotoxicity studies are presented in Tables 1, 2 and Fig. 1. Fig. 1 shows the % cell viability of the cell lines after treatment with carboranyl glycoconjugates 15, 17, 19 and 21 those contain three and six glucose and galactose moieties respectively.

**Table 1:** IC_50_ values (μM) of carboranyl glycoconjugates

Compounds	Cell lines		
	HaCaT (normal epidermal cell line)	MCF-7 (breast cancer cell line)	A431 (skin cancer cell line)
15	80.2	49.9	68.7
17	84.5	58.3	72.0
19	96.4	91.9	85.1
21	92.9	89.5	86.8

**Table 2:** % Inhibition of cell lines by carboranyl glycoconjugates at 100 μM concentration

Compounds	HaCaT (normal epidermal cell line)	MCF-7 (breast cancer cell line)	A431 (skin cancer cell line)
15	44.8	81.3	76.3
17	42.8	83.0	78.6
19	33.6	44.3	50.9
21	38.8	48.4	37.1

All glycoconjugates of *o*-carborane were found to be relatively nontoxic and they display lowest cytotoxicities towards normal skin epidermal cell line HaCaT. The IC_50_ values of compounds 15 and 17 (with three glucose and galactose units) were found to be between 80–85 μM, whereas the IC_50_ values for compounds 19 and 21 (with six glucose and galactose units) were found to be between 92-94 μM. Low cytotoxicity of carboranyl glycoconjugates towards healthy cells is essential for their use as a good chemotherapy agents and effective boron neutron capture therapy (BNCT) agents as well. The anti-cancer activity of carboranyl glycoconjugates can be further assessed from the plot of % cell viability *vs.* concentration (Fig. 1). The highest % cell viability was observed for normal skin epidermal cell line HaCaT followed by skin cancerous cell line A431 (Fig. 1). Breast cancerous cell line MCF-7 showed the lowest % cell viability indicating that carboranyl glycoconjugates are most cytotoxic towards these cells.

The anti-cancer effects of carboranyl glycoconjugates can further be assessed form the inhibition of cell proliferation shown in Table 2. Administration of 100 μM concentrations of compounds 15 and 17 (with three glucose and galactose units with *o*-carborane) inhibited up to 83% of MCF-7 breast cancer cells and up to 79% A431 skin cancer cells. Whereas administration of similar concentrations of compounds 19 and 21 (with six glucose and galactose units with *o*-carborane) inhibited only up to 48% of MCF-7 breast cancer cells and only up to 50% A431 skin cancer cells. However, all four carboranyl glycoconjugates were found to be relatively nontoxic towards HaCaT normal epidermal cells and only up to 34–45% inhibition was observed at 100 μM concentration. The IC_50_ value of the most commonly used cancer drug, cisplatin has been reported to be between 6 μg mL^−1^ to 9 μg mL^−1^ (20 to 30 μM) for A431 skin cancer cells and 6 μg mL^−1^ (20 μM) for MCF-7 breast cancer cells.^45–47^ It is more than two fold higher than the IC_50_ values observed for carboranyl glycoconjugates 15 and 17 (with three glucose and galactose units with *o*-carborane). The IC_50_ of cisplatin is more than three fold higher than the IC_50_ values observed for carboranyl glycoconjugates 19 and 21 (with three glucose and galactose units with *o*-carborane). However, IC_50_ values of carboranyl glycoconjugates are found to be more than three fold lower than the IC_50_ of cisplatin (25.5 μM) observed for HaCaT normal epidermal cell.^48^ Thus carboranyl glycoconjugates 15 and 17 (with three glucose and galactose units with *o*-carborane) are found to be more cytotoxic to cancer cells than normal body cells. It has also been observed that increasing the number of sugar moieties decreases the cytotoxicity of carboranyl glycoconjugates. Less cytotoxicity of dendritic carboranyl glycoconjugates towards normal body cells could make them ideal candidates for their use as dual mode candidates for cancer therapy *i.e.* as chemotherapy agents and boron neutron capture (BNCT) agents. It is expected that macromolecular and dendritic carborane–sugar conjugates 15, 17, 19 and 21 could show preferential accumulation in cancer cells due to the enhanced permeability and retention (EPR) effect.^14–16^ In addition, the over expression carbohydrate specific receptor proteins in cancer cell would also facilitate their uptake into the cancer cells.^19–22,49^ The trend of cytotoxicity between compound 15 and 17 indicates no significant difference and thus the specific role of sugar residue in binding or affinity based cytotoxicity can be ruled out. In addition, the study reveals that the sugar moieties just help to enhance the polarity and water solubility of conjugates and increasing the polarity beyond certain point as in molecules 19 and 21 is detrimental towards their cytotoxicity. It highlights the importance of amphiphilicity rather than enhanced water solubility for the better cell permeability and anti-cancer activity.^50^

## Conclusion

In summary, we have synthesized dendritic and water-soluble carboranyl glycoconjugates containing three and six glucose and galactose moieties *via* Cu(i)-catalyzed Huisgen-type azide alkyne cycloaddition reaction. Carboranyl glycoconjugates containing three glucose and galactose moieties were found to be partially water soluble. But increasing the number of glucose and galactose moieties to six made them completely water soluble. Evaluation of cytotoxicities of newly synthesized carboranyl glycoconjugates were carried out using two cancer cell lines, MCF-7 (breast cancer cells) and A431 (skin cancer cells). Their cytotoxicities were compared with HaCaT normal epidermal cells. The IC_50_ values obtained for cancer cells were found to be significantly higher for carboranyl glycoconjugates containing three glucose and galactose moieties (15 and 17). They could inhibit 76–83% cancer cells at 100 μM concentrations. Increasing the glucose and galactose moieties to six reduced their cytotoxicities towards cancer cells. However, all carboranyl glycoconjugates showed significantly lower cytotoxicities towards HaCaT normal epidermal cells. Even their cytotoxicities towards HaCaT normal epidermal cells were found to be significantly lower than the common chemotherapy agent cisplatin. Such property of carboranyl glycoconjugates is essential for their use as chemotherapy agent and BNCT agent as well.

## Experimental

### General information

All experiments were carried out in oven-dried flasks under dry argon/nitrogen. Solvents were distilled under nitrogen from an appropriate drying agent. Reagents were purchased and were used without further purification. All compounds were purified by column chromatography on silica gel (60–120 mesh, Spectrochem, India). Yield of the products refer to analytically pure samples. ^1^H and ^13^C NMR spectra were recorded on Bruker Fourier Transform multinuclear NMR spectrometer at 400 MHz and 100 MHz respectively. Chemical shifts are reported relative to TMS (1H: *δ* = 0.00 ppm), CDCl_3_ (^13^C: *δ* = 77.0 ppm) and coupling constants are given in Hz. All ^13^C spectra are proton-decoupled. ^11^B NMR spectra are proton-decoupled and recorded at 128 MHz relative to BF_3_·Et_2_O. Accurate mass measurements were performed for compounds using Waters QTOF micro with lock spray instrument by both ES or APCI ion source (in the positive or negative mode) and MALDI-TOF mass spectral analyses were performed with Shimadzu Biotech Axima Confidence spectrometer using dithranol as matrix.

### Cytotoxicity test

3-(4,5-Dimethylthiazol-2-yl)-2,5-diphenyl tetrazolium bromide (MTT), Dulbecco's modified Eagle's medium (DMEM), fetal bovine serum (FBS), antibiotic solution (penicillin–streptomycin) were purchased from Sigma Aldrich (Mumbai, India) and 96 wells plates were purchased from Tarsons, India. MCF-7 (breast carcinoma), HaCaT (normal skin epidermal cell) and A431 (skin carcinoma) cell lines were purchased from NCCS, Pune, India. The cells were seeded in 96 well plates at the density of 3000 cells per well based on the doubling time in the presence of 200 μL DMEM (Dulbecco's modified Eagle's medium) supplemented with 10% FBS and 1% penicillin–streptomycin solution and incubated for 24 h in an incubator containing 5% CO_2_ at 37 °C. After 24 h of seeding, the existing media was removed and replaced by fresh media along with various concentrations of compounds (15, 17, 19 and 21) such as 10, 20, 40, 60, 80, 90 and 100 μM and incubated for 24 h at 37 °C, 5% CO_2_. To detect the cell viability, MTT working solution was prepared from a stock solution of 5 mg mL^−1^ in growth medium without FBS to the final concentration of 0.8 mg mL^−1^. 100 μL of MTT solution was added and incubated for 4 h. After 4 h of incubation, the MTT solution was discarded and 100 μL of DMSO solvent was added to each well to dissolve the formazan produced under dark followed by an incubation of 15 min. The optical density of the formazan product was read at 595 nm in a micro plate reader (Perkin Elmer, Waltham, MS, USA).

### Synthesis and analytical data of compounds

#### Compound 2

To a solution of *p*-iodo anisole 1 (2 g, 8.54 mmol) in 1 : 1 mixture of dry THF (10 mL) and dry Et_3_N (10 mL), trimethylsilylacetylene (1.5 mL) was added under argon atmosphere followed by addition of Pd(PPh_3_)_2_Cl_2_ (144 mg, 0.02 mmol), CuI (79 mg, 0.41 mmol) and was stirred at room temperature for 6 hour. The progress of the reaction was monitored by TLC. The reaction mixture was filtered over short silica pad. After evaporating the solvent crude product was purified by silica gel column chromatography using 1% ethyl acetate in hexane to obtain 1.7 g of the pure product 2. Yield: 97%.

#### Compound 3

To a solution of 2 (1.6 g, 7.84 mmol) in 1 : 1 mixture of dry THF (10 mL) & dry methanol (10 mL), K_2_CO_3_ (1.62 g, 11.76 mmol) was added under argon atmosphere and stirred at room temperature for 3 hours. The progress of the reaction was monitored by TLC. After completion of the reaction, the reaction mixture was filtered over short silica pad. The solvent was evaporated and the crude product was purified by silica gel column chromatography using 1% ethyl acetate in hexane to obtain 1 g of the pure product 3. Yield: 97%.

#### Compound 4

Decaborane (1.1 g, 9.08 mmol) was refluxed at 95 °C in dry acetonitrile (10 mL) for two hours under argon atmosphere and compound 2 (1 g, 7.57 mmol) was added to it in toluene (5 mL) at room temperature and refluxed at 95 °C for 17 h. The progress of the reaction was monitored by TLC. Excess decaborane was quenched by adding 2 mL of methanol and concentrated to get the crude product which was purified by silica gel chromatography using 2% ethyl acetate in hexane to obtain 400 mg of pure compound 4 as a colorless solid. Yield: 22%. Mp: 114 °C. ^1^H NMR (400 MHz, CDCl_3_): *δ* 7.35 (d, 2H, *J* = 8 Hz, Ar-H), 6.75 (d, 2H, *J* = 8 Hz, Ar-H), 3.80 (br, s, 1H, cage-H), 3.73 (s, 3H, OCH_3_–H); ^13^C {^1^H} NMR (100 MHz, CDCl_3_): *δ* 160.7 (Ar-C), 129.2 (Ar-C), 125.5 (Ar-C), 114.0 (Ar-C), 60.9 (cage-C), 55.4 (OCH_3_–C); ^11^B {^1^H} NMR: −1.9, −4.7, −9.1, −10.6, −11.4, −12.7; IR (KBr): 3003, 2931, 2598 (B–H), 1610, 1516, 1268 cm^−1^; ES-MS (*m*/*z*): calc. for C_9_H_17_B_10_O: 249.34, found 249.2 [M–H]^+^.

#### Compound 5

BBr_3_ (3 mL, 3 mmol, 1 M in CH_2_Cl_2_) was added drop wise to a solution of 4 (200 mg, 0.84 mmol) in dry CH_2_Cl_2_ (8 mL) at 0 °C and stirred at room temperature for 15 hour. The progress of the reaction was monitored by TLC. After completion, the reaction mixture was quenched slowly with water (10 mL) at 0 °C. Then the crude product was extracted with CH_2_Cl_2_ (3 × 20 mL) and the combined organic layer were dried over anhydrous sodium sulfate and concentrated to afford the crude product, which was purified by silica gel chromatography using 9% ethyl acetate in hexane to obtain 180 mg of pure product 5 as a colorless solid. Yield: 95%. Mp: 105 °C. ^1^H NMR (400 MHz, CDCl_3_) *δ*: 7.41 (d, 2H, *J* = 8 Hz, Ar-H), 6.79 (d, 2H, *J* = 8 Hz, Ar-H), 5.03 (s, 1H, OH–H), 3.83 (br s, 1H, cage-H); ^13^C {^1^H} NMR (100 MHz, CDCl_3_): *δ* 156.8 (Ar-C), 129.5 (Ar-C), 125.9 (Ar-C), 115.5 (Ar-C), 60.9 (cage-C); ^11^B {^1^H} NMR: −1.8, −4.6, −9.1, −10.6, −11.4, −12.7; IR (KBr): 3230, 3064, 2961, 2597 (B–H), 1614, 1510, 1248 cm^−1^; ES-MS (*m*/*z*): calc. for C_8_H_15_B_10_O: 235.31, found 235.2 [M–H]^+^.

#### Compound 7

Compound 5 (100 mg, 0.44 mmol) was solubilized in 20 mL of dry acetone under argon. To this solution compound 6 (193 mg, 0.577 mmol) and K_2_CO_3_ (183 mg, 1.33 mmol) were added, and the reaction mixture was refluxed at 90 °C for 17 hours. The progress of the reaction was monitored by TLC. The reaction mixture was filtered through short silica pad, and the solvent was evaporated. The crude product was purified by silica gel chromatography using 9% ethyl acetate in hexane to obtain 200 mg of pure product 7 as a colorless solid. Yield: 92%. Mp: 90 °C. ^1^H NMR (400 MHz, CDCl_3_) *δ*: 7.35 (d, 2H, *J* = 8 Hz, Ar-H), 6.82 (d, 2H, *J* = 8 Hz, Ar-H), 6.74 (s, 2H, Ar-H), 4.93 (s, 2H, OCH_2_–H), 4.69 (d, 4H, *J* = 2.4 Hz, OCH_2_–H), 4.66 (d, 2H, *J* = 2.4 Hz, OCH_2_–H), 3.81 (s, 1H, cage-H), 2.40 (dd, 3H, *J* = 2.4 Hz, alkyne-H); ^13^C {^1^H} NMR (100 MHz, CDCl_3_) *δ*: 159.7 (Ar-C), 151.8 (Ar-C), 137.0 (Ar-C), 132.4 (Ar-C), 129.3 (Ar-C), 125.9 (Ar-C), 114.9 (Ar-C), 107.8 (Ar-C), 79.0 (alkyne-C), 78.3 (alkyne-C), 75.9 (alkyne-C), 75.3 (alkyne-C), 70.0 (OCH_2_–C), 60.9 (cage-C), 60.3 (OCH_2_–C), 57.1 (OCH_2_–C); ^11^B {^1^H} NMR: −1.8, −4.6, −9.0, −10.6, −11.3, −12.5; IR (KBr): 2926, 2595 (B–H), 2124, 1605, 1511, 1254 cm^−1^; HRMS (*m*/*z*): calc. for C_24_H_27_B_10_O_4_: 489.2840, found 489.3035 [M–H]^+^.

#### Compound 8

To a solution of 3 (700 mg, 5.3 mmol) in 15 mL of Et_3_N, *p*-iodo anisole (992 mg, 4.2 mmol) was added under argon atmosphere followed by Pd(PPh_3_)Cl_2_ (11.2 mg, 0.16 mmol) and CuI (61 mg, 0.32 mmol) were added and refluxed at 80 °C for 5 hour. The progress of the reaction was monitored by TLC. After completion of the reaction the excess acetonitrile was removed and the crude product was purified by silica gel chromatography using 3% ethyl acetate in hexane to obtain 765 mg of the pure product 8 as colorless solid. Yield: 61%. Mp: 142 °C. ^1^H NMR (400 MHz, CDCl_3_) *δ*: 7.37 (d, 4H, *J* = 8 Hz, Ar-H), 6.78 (d, 4H, *J* = 8 Hz, Ar-H), 3.73 (s, 6H, OCH_3_–H); ^13^C {^1^H} NMR (100 MHz, CDCl_3_) *δ*: 159.4 (Ar-C), 132.8 (Ar-C), 115.7 (Ar-C), 113.9 (Ar-C), 87.9 (alkyne-C), 55.3 (OCH_3_–C); IR (KBr): 2969, 2038, 1606, 1519, 1285, 1106 cm^−1^.

#### Compound 9

Decaborane (462 mg, 3.77 mmol) was refluxed at 95 °C in acetonitrile (5 mL) for two hour under argon atmosphere and to it compound 8 (600 mg, 2.52 mmol) dissolved in toluene (5 mL) was added at room temperature and was refluxed at 95 °C for 17 hour. The progress of the reaction was monitored by TLC. Excess decaborane was quenched using 2 mL of methanol and concentrated to get the crude product which was purified by silica gel chromatography using 3% ethyl acetate in hexane to obtain 300 mg of pure product 9 as a colorless solid. Yield: 34%. Mp: 144 °C. ^1^H NMR (400 MHz, CDCl_3_) *δ*: 7.37 (d, 4H, *J* = 8 Hz, Ar-H), 6.66 (d, 4H, *J* = 8 Hz, Ar-H), 3.75 (s, 6H, OCH_3_–H); ^13^C {^1^H} NMR (100 MHz, CDCl_3_) *δ*: 160.7 (Ar-C), 132.1 (Ar-C), 123.1 (Ar-C), 113.5 (Ar-C), 85.9 (cage-C), 55.27 (OCH_3_–C); ^11^B {^1^H} NMR: −2.8, −9.1, −10.8; IR (KBr): 2962, 2933, 2592 (B–H), 1605, 1512, 1259 cm^−1^; HRMS (*m*/*z*): calc. for C_16_H_23_B_10_O_4_: 357.2629, found 357.2718 [M–H]^+^.

#### Compound 10

BBr_3_ (6.1 mL, 6.1 mmol, 1 M in CH_2_Cl_2_) was added drop wise to a solution of 9 (300 mg, 0.86 mmol) in CH_2_Cl_2_ (15 mL) at 0 °C and stirred at room temperature for 15 hour. The progress of the reaction was monitored by TLC. After completion, the reaction mixture was quenched slowly with water (20 mL) at 0 °C. Then the crude product was extracted with CH_2_Cl_2_ (3 × 30 mL) and the combined organic layer were dried over sodium sulfate. Removal of the solvent afforded the crude product, which was purified by silica gel chromatography using 20% ethyl acetate in hexane to obtain 260 mg of pure product 10 as a colorless solid. Yield: 94%. Mp: 140 °C. ^1^H NMR (400 MHz, CD_3_CN) *δ*: 7.37 (d, 4H, *J* = 8 Hz, Ar-H), 6.63 (d, 4H, *J* = 8 Hz, Ar-H), 7.33 (s, 2H, OH–H); ^13^C {^1^H} NMR (100 MHz, CD_3_CN) *δ*: 159.3 (Ar-C), 133.1 (Ar-C), 122.5 (Ar-C), 115.4 (Ar-C), 87.4 (cage-C); ^11^B {^1^H} NMR: −3.3, −9.2, −11.2; IR (KBr): 2926, 2853, 2602 (B–H), 1611, 1513, 1252 cm^−1^; ES-MS (*m*/*z*): calc. for C_14_H_19_B_10_O_2_: 327.4, found 327.2 [M–H]^+^.

#### Compound 11

Compound 10 (120 mg, 0.38 mmol) was solubilized in 25 mL of dry acetone under argon. To this solution compound 6 (326 mg, 0.98 mmol) and K_2_CO_3_ (312 mg, 2.26 mmol) were added and the reaction mixture was refluxed at 90 °C for 17 hours. The progress of the reaction was monitored by TLC. The reaction mixture was filtered through short silica pad and the solvent was concentrated. The crude product was purified by silica gel chromatography using 20% ethyl acetate in hexane to obtain 240 mg of pure product 11 as a colorless solid. Yield: 76%. Mp: 103 °C. ^1^H NMR (400 MHz, CDCl_3_) *δ*: 7.28 (d, 4H, *J* = 12 Hz, Ar-H), 6.69 (s, 4H, Ar-H), 6.64 (d, 4H, *J* = 12 Hz, Ar-H), 4.85 (s, 4H, OCH_2_–H), 4.65 (dd, 12H, *J* = 2.4 Hz, OCH_2_–H), 2.38 (t, 2H, *J* = 2.4 Hz, alkyne-H), 2.35 (t, 4H, *J* = 2.4 Hz, alkyne-H); ^13^C {^1^H} NMR (100 MHz, CDCl_3_) *δ*: 159.8 (Ar-C), 151.7 (Ar-C), 136.9 (Ar-C), 132.3 (Ar-C), 132.1 (Ar-C), 123.5 (Ar-C), 114.4 (Ar-C), 107.7 (Ar-C), 85.7 (cage-C), 79.0 (alkyne-C), 78.3 (alkyne-C), 76.0 (alkyne-C), 75.3 (alkyne-C), 69.7 (OCH_2_–C), 60.3 (OCH_2_–C), 57.0 (OCH_2_–C); ^11^B {^1^H} NMR: −2.3, −10.4; IR (KBr): 2926, 2589 (B–H), 2125, 1602, 1509, 1255 cm^−1^; ES-MS (*m*/*z*): calc. for C_46_H_44_B_10_O_8_: 834.39, found 834.40 [M]^+^; 852.43 [M + H_2_O]^+^.

### General procedure for preparation of dendritic glycoconjugates using click reaction

The alkynyl core and the sugar azide were dissolved in dry THF and then sodium ascorbate, CuSO_4_·5H_2_O and water were added to it. The reaction mixture was stirred at room temperature for 24 hour. The reaction was monitored by TLC and after completion of the reaction the mixture was cooled using ice bath and ice cold water was added to it. The precipitate formed was filtered through vacuum filtration to get the crude compound which was purified by neutral alumina column chromatography.

#### Compound 14

The alkynyl core 7 (500 mg, 1.02 mmol), glucosyl azide 12 (1409 mg, 3.37 mmol), THF (5 mL), CuSO_4_·5H_2_O (25 mg, 0.1 mmol), sodium ascorbate (200 mg, 1 mmol), and water (5 mL). Purification: neutral alumina column chromatography using 2% methanol in ethyl acetate. Pure product: 920 mg (colorless solid). Yield: 52.5%. Mp: 92 °C. ^1^H NMR (400 MHz, CDCl_3_) *δ*: 7.81 (s, 2H, alkene-H), 7.76 (s, 1H, alkene-H), 7.37 (d, 2H, Ar-H, *J* = 12 Hz), 6.84 (d, 2H, *J* = 8 Hz, Ar-H), 6.76 (s, 2H, Ar-H), 5.15–5.06 [m, 9H (Glu-H); 2H (OCH_2_–H)], 5.02–4.88 [m, 3H (Glu-H); 6H (OCH_2_–H)], 4.58–4.44 [m, 3H (Glu-H); 6H (CH_2_–H)], 4.23–3.86 [m, 3H (Glu-H); 6H (CH_2_–H)], 3.68–3.60 [m, 3H (Glu-H)], 2.0, 1.95, 1.91, 1.86 (4s, 36H, 12 CH_3_); ^13^C {^1^H}NMR (100 MHz, CDCl_3_) *δ*: 170.6, 170.1, 169.5, 169.3 (4× OAc-C), 159.7 (Ar-C), 152.4 (Ar-C), 144.4 (alkene C), 137.5 (Ar-C), 132.4 (Ar-C), 129.3 (Ar-C), 125.9 (Ar-C), 124.6 (alkene C), 114.9 (Ar-C), 107.1 (Ar-C), 100.5, 100.4, 72.5, 72.4, 72.0, 71.9, 70.8, 70.8 (Glu-Cs), 70.8 (OCH_2_–C), 68.3, 68.2 (Glu-C), 67.6 (OCH_2_–C), 63.4 (OCH_2_–C), 61.9 (CH_2_–C), 61.8, 61.7 (Glu-C), 61.0 (cage-C), 50.0 (CH_2_–C), 20.7, 20.6, 20.5 (4× CH_3_–C); ^11^B {^1^H} NMR: −1.9, −4.6, −9.1, −10.7; IR (KBr): 2927, 2853, 2596 (B–H), 1755 (ester-CO), 1665, 1599, 1511, 1380, 1228, 1041 cm^−1^; HRMS (*m*/*z*): calc. for C_72_H_97_B_10_N_9_O_34_: 1741.7068, found 1741.7218 [M]^+^.

#### Compound 16

The alkynyl core 7 (500 mg, 1 mmol), galactose azide 13 (1409 mg, 3.4 mmol), THF (5 mL), CuSO_4_·5H_2_O (25 mg, 0.1 mmol), sodium ascorbate (200 mg, 1 mmol), and water (5 mL). Purification: neutral alumina column chromatography using 2% methanol in ethyl acetate. Pure product: 900 mg (colorless solid).Yield: 51.4%. Mp: 96 °C. ^1^H NMR (400 MHz, CDCl_3_) *δ*: 7.79 (s, 2H, alkene-H), 7.78 (s, 1H, alkene-H), 7.37 (d, 2H, Ar-H, *J* = 12 Hz), 6.84 (d, 2H, *J* = 8 Hz, Ar-H), 6.77 (s, 2H, Ar-H), 5.34–5.04 [m, 9H (Gal-H); 2H (OCH_2_–H)], 4.96–4.89 [m, 3H (Gal-H); 6H (OCH_2_–H)], 4.58–4.40 [m, 3H (Gal-H); 6H (CH_2_–H)], 4.24–4.02 [m, 3H (Gal-H); 6H (CH_2_–H)], 3.92–3.84 [m, 3H (Gal-H)], 2.07, 1.97, 1.90, 1.86 (4s, 36H, 12 CH_3_); ^13^C {^1^H} NMR (100 MHz, CDCl_3_) *δ*: 170.4, 170.1, 170.0, 169.5 (4× OAc-C), 159.7 (Ar-C), 152.5 (Ar-C), 144.5 (alkene C), 137.5 (Ar-C), 132.4 (Ar-C), 129.4 (Ar-C), 125.8 (Ar-C), 124.6 (alkene C), 114.9 (Ar-C), 107.0 (Ar-C), 100.9, 100.8, 70.9, 70.8, 70.6, 70.5 (Gal-Cs), 70.4 (OCH_2_–C), 68.4, 68.3 (Gal-Cs), 67.6 (OCH_2_–C), 67.0, 66.9 (Gal-Cs), 63.0 (OCH_2_–C), 62.5 (CH_2_–C), 61.1, 61.0 (Gal-C), 61.0 (cage-C), 50.0 (CH_2_–C), 20.7, 20.6, 20.5, 20.4 (4× CH_3_–C); ^11^B {^1^H} NMR: −2.09, −4.62, −9.25, −10.92; IR (KBr): 2924, 2853, 2597 (B–H), 1750 (ester CO), 1665, 1512, 1383, 1225, 1049 cm^−1^; HRMS (*m*/*z*): calc. for C_72_H_97_B_10_N_9_O_34_: 1741.7068, found 1741.7212 [M]^+^.

#### Compound 18

The alkynyl core 11 (500 mg, 0.6 mmol), glucosyl azide 12 (1.65 g, 3.96 mmol), THF (8 mL), CuSO_4_·5H_2_O (65 mg, 0.261 mmol), sodium ascorbate (517 mg, 2.61 mmol), and water (8 mL). Purification: neutral alumina column chromatography using 3% methanol in ethyl acetate. Pure product: 1090 mg (colorless solid). Yield: 54.5%. Mp: 110 °C. ^1^H NMR (400 MHz, CDCl_3_) *δ*: 7.81 (s, 4H, alkene-H), 7.76 (s, 2H, alkene-H), 7.32 (d, 4H, Ar-H, *J* = 8 Hz), 6.73 (s, 4H, Ar-H), 6.69 (d, 4H, *J* = 8 Hz, Ar-H), 5.13–5.04 [m, 18H (Glu-H); 4H (OCH_2_–H)], 5.02–4.81 [m, 6H (Glu-H); 12H (OCH_2_–H)], 4.58–4.45 [m, 6H (Glu-H); 12H (CH_2_–H)], 4.24–3.88 [m, 6H (Glu-H); 12H (CH_2_–H)], 3.70–3.55 [m, 6H (Glu-H)], 2.0, 1.95, 1.91, 1.84 (4s, 72H, 24 CH_3_); ^13^C {^1^H}NMR (100 MHz, CDCl_3_) *δ*: 170.6, 170.1, 169.4, 169.3 (4× OAc-C), 159.8 (Ar-C), 152.4 (Ar-C), 144.3 (alkene C), 137.5 (Ar-C), 132.2 (Ar-C), 124.9 (Ar-C), 124.5 (Ar-C), 123.5 (alkene C), 114.4 (Ar-C), 107.2 (Ar-C), 100.5, 100.4 (Glu-Cs), 85.9 (cage-C), 72.5, 72.4, 71.9, 71.8 (Glu-Cs), 69.9 (OCH_2_–C), 68.3, 68.2 (Glu-C), 67.6 (OCH_2_–C), 63.0 (OCH_2_–C), 61.8, 61.7 (Glu-C), 49.9 (CH_2_–C), 20.7, 20.6, 20.5 (4× CH_3_–C); ^11^B {^1^H} NMR: −2.98, −11.23; IR (KBr): 2925, 2853, 2591 (B–H), 1754 (ester CO), 1604, 1510, 1377, 1227, 1041 cm^−1^; HRMS (*m*/*z*): calc. for C_142_H_183_B_10_N_18_O_68_: 3338.2562, found: 3338.2348 [M + H]^+^.

#### Compound 20

The alkynyl core 17 (500 mg, 0.6 mmol), galactose azide 13 (1652 mg, 3.96 mmol), THF (8 mL), CuSO_4_·5H_2_O (65 mg, 0.261 mmol), sodium ascorbate (517 mg, 2.61 mmol), and water (8 mL). Purification: neutral alumina column chromatography using 3% methanol in ethyl acetate. Pure product: 1050 mg (colorless solid).Yield: 52.5%. Mp: 105 °C. ^1^H NMR (400 MHz, CDCl_3_) *δ*: 7.80 (s, 4H, alkene-H), 7.77 (s, 2H, alkene-H), 7.32 (d, 4H, Ar-H, *J* = 8 Hz), 6.74 (s, 4H, Ar-H), 6.68 (d, 4H, *J* = 8 Hz, Ar-H), 5.34–5.02 [m, 18H (Gal-H); 4H (OCH_2_–H)], 4.96–4.79 [m, 6H (Gal-H); 12H (OCH_2_–H)], 4.61–4.39 [m, 6H (Gal-H); 12H (CH_2_–H)], 4.25–3.82 [m, 6H (Gal-H); 12H (CH_2_–H)], 3.75–3.57 [m, 6H (Gal-H)], 2.07, 1.97, 1.89, 1.85 (4s, 72H, 24 CH_3_); ^13^C {^1^H} NMR (100 MHz, CDCl_3_) *δ*: 170.4, 170.1, 170.0, 169.5 (4× OAc-C), 159.7 (Ar-C), 152.4 (Ar-C), 144.5 (alkene C), 137.5 (Ar-C), 132.2 (Ar-C), 124.9 (Ar-C), 124.6 (Ar-C), 123.5 (alkene C), 114.4 (Ar-C), 107.1 (Ar-C), 100.9, 100.8 (Gal-Cs), 85.8 (cage-C), 70.9, 70.8, 70.6, 70.5 (Gal-Cs), 69.9 (OCH_2_–C), 68.4, 68.3 (Gal-Cs), 67.5 (OCH_2_–C), 67.9, 66.9 (Gal-Cs), 66.5 (OCH_2_–C), 63.0 (OCH_2_–C), 61.7 (CH_2_–C), 61.2, 61.1 (Gal-C), 50.0 (CH_2_–C), 20.8, 20.7, 20.6, 20.5 (4× CH_3_–C); ^11^B {^1^H} NMR: −1.80, −10.21; IR (KBr): 2959, 2853, 2591 (B–H), 1747 (ester CO), 1635, 1509, 1370, 1224, 1050 cm^−1^; HRMS (*m*/*z*): calc. for C_142_H_183_B_10_N_18_O_68_: 3338.2562, found: 3338.2546 [M + H]^+^.

### General procedure for deacetylation reaction

The acetylated carborane–sugar conjugates were dissolved in dry methanol, and a solution of sodium methoxide in anhydrous methanol was added and reaction mixture was stirred at room temperature (45 min to 3 h). The progress of the reaction was monitored by TLC and when starting material was disappeared the reaction mixture was neutralized by the addition of Amberlite IR-120 (H^+^-form) ion exchange resin (pre-washed with anhydrous methanol). The reaction mixture was then filtered over a cotton plug and concentrated under reduced pressure to afford carborane–sugar conjugates without acetyl moieties.

#### Compound 15

Acetylated derivative 14 (300 mg, 0.172 mmol), sodium methoxide (5 mg), anhydrous methanol (10 mL). Room temperature, 45 min. Pure product: 195 mg (colorless solid). Yield: 91%. Mp: 105 °C. ^1^H NMR (400 MHz, MeOD) *δ*: 8.22 (s, 2H, alkene-H), 8.01 (s, 1H, alkene-H), 7.51 (d, 2H, Ar-H, *J* = 8 Hz), 7.0 (d, 2H, *J* = 8 Hz, Ar-H), 6.93 (s, 2H, Ar-H), 5.22–5.0 [m, 9H (Glu-H); 2H (OCH_2_–H)], 4.74–4.55 [m, 3H (Glu-H); 6H (OCH_2_–H)], 4.35–4.06 [m, 3H (Glu-H); 6H (CH_2_–H)], 3.91–3.61 [m, 3H (Glu-H); 6H (CH_2_–H)], 3.39–3.15 [m, 3H (Glu-H)]; ^13^C {^1^H}NMR (100 MHz, MeOD) *δ*: 159.8 (Ar-C), 152.3 (Ar-C), 143.2 (alkene C), 136.8 (Ar-C), 133.3 (Ar-C), 128.8 (Ar-C), 126.0 (Ar-C), 125.4 (alkene C), 114.7 (Ar-C), 107.2 (Ar-C), 103.2, 76.6, 76.5, 73.5 (Glu-Cs), 70.1 (OCH_2_–C), 69.6 (Glu-C), 67.7 (OCH_2_–C), 65.3 (OCH_2_–C), 62.2 (CH_2_–C), 61.3 (Glu-C), 61.2 (cage-C), 50.3 (CH_2_–C); ^11^B {^1^H} NMR: −2.8, −9.4, −11.08; IR (KBr): 3423, 2928, 2853, 2594 (B–H), 1637, 1602, 1510, 1381, 1250, 1075 cm ^−1^; HRMS (*m*/*z*): calc. for C_48_H_72_B_10_N_9_O_22_: 1236.5722, found 1236.5787 [M–H]^+^.

#### Compound 17

Acetylated derivative 16 (200 mg, 0.114 mmol), sodium methoxide (3 mg), anhydrous methanol (10 mL). Room temperature, 45 min. Pure product: 120 mg (colorless solid). Yield: 84%. Mp: 100 °C. ^1^H NMR (400 MHz, MeOD) *δ*: 8.23 (s, 2H, alkene-H), 7.78 (s, 1H, alkene-H), 7.51 (d, 2H, Ar-H, *J* = 8 Hz), 7.0 (d, 2H, *J* = 8 Hz, Ar-H), 6.49 (s, 2H, Ar-H), 5.30–5.0 [m, 9H (Gal-H); 2H (OCH_2_–H)], 4.74–4.45 [m, 3H (Gal-H); 6H (OCH_2_–H)], 4.35–4.95 [m, 3H (Gal-H); 6H (CH_2_–H)], 3.92–3.64 [m, 3H (Gal-H); 6H (CH_2_–H)], 3.59–3.20 [m, 3H (Gal-H)]; ^13^C {^1^H}NMR (100 MHz, MeOD) *δ*: 159.8 (Ar-C), 152.3 (Ar-C), 143.3 (alkene C), 136.7 (Ar-C), 133.2 (Ar-C), 128.8 (Ar-C), 126.0 (Ar-C), 125.4 (alkene C), 114.7 (Ar-C), 107.1 (Ar-C), 103.8, 75.4, 73.4 (Gal-Cs), 70.9 (OCH_2_–C), 69.6, 68.9 (Gal-Cs), 67.7 (OCH_2_–C), 65.5 (OCH_2_–C), 62.2 (CH_2_–C), 61.1 (CH_2_–C), 50.4 (CH_2_–C); ^11^B {^1^H} NMR: −2.78, −9.32, −10.95; IR (KBr): 3415, 2921, 2880, 2591 (B–H), 1638, 1601, 1509, 1381, 1252, 1076 cm^−1^; HRMS (*m*/*z*): calc. for C_48_H_72_B_10_N_9_O_22_: 1236.5722, found 1236.5794 [M–H]^+^.

#### Compound 19

Acetylated derivative 18 (300 mg, 0.089 mmol), sodium methoxide (8 mg), anhydrous methanol (10 mL). Room temperature, 3 h. Pure product: 180 mg (colorless solid). Yield: 86%. Mp: 140 °C. ^1^H NMR (400 MHz, MeOD) *δ*: 8.17 (s, 4H, alkene-H), 7.94 (s, 2H, alkene-H), 7.46 (d, 4H, Ar-H, *J* = 8 Hz), 6.84 (br s, 4H, Ar-H), 6.82 (s, 4H, Ar-H), 5.16–5.04 [m, 18H (Glu-H); 4H (OCH_2_–H)], 4.78–4.47 [m, 6H (Glu-H); 12H (OCH_2_–H)], 4.40–4.16 [m, 6H (Glu-H); 12H (CH_2_–H)], 4.07–3.59 [m, 6H (Glu-H); 12H (CH_2_–H)], 3.42–3.12 [m, 6H (Glu-H)]; ^13^C {^1^H} NMR (100 MHz, MeOD) *δ*: 162.2, 152.2 (Ar-C), 143.2 (alkene C), 136.7 (Ar-C), 132.2 (Ar-C), 125.5 (Ar-C), 125.4 (Ar-C), 123.0 (alkene C), 114.4 (Ar-C), 107.2 (Ar-C), 103.2 (Glu-C), 86.3 (cage-C), 76.4, 76.5, 73.5, 70.1 (Glu-Cs), 69.5 (OCH_2_–C), 67.2, (Glu-C), 65.5 (OCH_2_–C), 62.2 (OCH_2_–C), 61.3 (Glu-C), 50.3 (CH_2_–C); ^11^B {^1^H} NMR: −6.6, −14.5; IR (KBr): 3415, 2921, 2880, 2591 (B–H), 1638, 1601, 1509, 1381, 1252, 1076 cm^−1^; HRMS (*m*/*z*): calc. for C_94_H_133_B_10_N_18_O_44_: 2327.9654, found: 2327.9685 [M–H]^+^.

#### Compound 21

Acetylated derivative 20 (180 mg, 0.053 mmol), sodium methoxide (5 mg), anhydrous methanol (10 mL). Room temperature, 3 h. Pure product: 115 mg (colorless solid). Yield: 93%. Mp: 130 °C. ^1^H NMR (400 MHz, D_2_O) *δ*: 7.86 (br s, 6H, alkene-H), 7.66 (br s, 4H, Ar-H), 6.74 (s, 4H, Ar-H), 6.42 (br s, 4H, *J* = 8 Hz, Ar-H), 4.60–4.16 [m, 18H (Gal-H); 4H (OCH_2_–H)], 4.06–3.77 [m, 6H (Gal-H); 12H (OCH_2_–H)], 3.61–3.42 [m, 6H (Gal-H); 12H (CH_2_–H)], 3.38–3.20 [m, 6H (Gal-H); 12H (CH_2_–H)], 3.10–2.97 [m, 6H (Gal-H)]; ^13^C {^1^H}NMR (100 MHz, D_2_O) *δ*: 159.7 (Ar-C), 151.6 (Ar-C), 142.7 (alkene C), 135.6 (Ar-C), 132.5 (Ar-C), 125.7 (Ar-C), 123.3 (Ar-C), 124.9 (alkene C), 114.4 (Ar-C), 107.1 (Ar-C), 103.0 (Gal-C), 81.6 (cage-C), 75.0, 72.7, 71.8, 71.8, 70.4 (Gal-Cs), 68.4 (OCH_2_–C), 67.8 (Gal-C), 65.7 (OCH_2_–C), 62.8, (OCH_2_–C), 61.8 (OCH_2_–C), 61.7 (CH_2_–C), 60.8 (Gal-C), 50.1 (CH_2_–C); ^11^B {^1^H} NMR: −5.05, −12.88; IR (KBr): 2959, 2853, 2591 (B–H), 1747 (ester CO), 1635, 1509, 1370, 1224, 1050 cm^−1^; HRMS (*m*/*z*): calc. for C_94_H_133_B_10_N_18_O_44_: 2327.9654, found: 2327.9714 [M–H]^+^.

## Conflicts of interest

The authors declare no conflict of interest.

## Supplementary Material

RA-010-D0RA07264H-s001
