# Supplementary material for: Preparation of dendritic carboranyl glycoconjugates as potential anticancer therapeutics
Source: RSC Adv. 2020 Sep 18;10(57):34764–74. doi: 10.1039/d0ra07264h (PMC9056817; doi:10.1039/d0ra07264h)
Supplement: RA-010-D0RA07264H-s001 [file RA-010-D0RA07264H-s001.pdf]

Electronic Supplementary Material (ESI) for RSC Advances.
This journal is © The Royal Society of Chemistry 2020

Supplementary information for:

Preparation of dendritic carboranyl glycoconjugates as potential anticancer therapeutics

Biswa Ranjan Swain,[a,b] Chandra Sekhara Mahanta,[a] Bibhuti Bhusan Jena,[a] Swaraj Kumar Beriha,[b] Bismita Nayak,[c] Rashmirekha Satapathy,[a*] and Barada P. Dash[b*]

[a]*Department of Chemistry, Ravenshaw University, Cuttack, Odisha, 753 003, India. E-mail: rashmi.satapathy@gmail.com; rashmi@ravenshawuniversity.ac.in*

[b]*Department of Chemistry, Siksha 'O' Anusandhan (Deemed to be University), Bhubaneswar, Odisha, 751030, India. E-mail: barada.dash@gmail.com; baradadash@soa.ac.in*

[c] *Department of Life Science, National Institute of Technology, Rourkela, Odisha, 769 008, India*

Contents **Page No.**

NMR spectra of compounds...2

Mass spectra of compounds...23

NMR Spectra

^{1}H NMR of Compound 4:

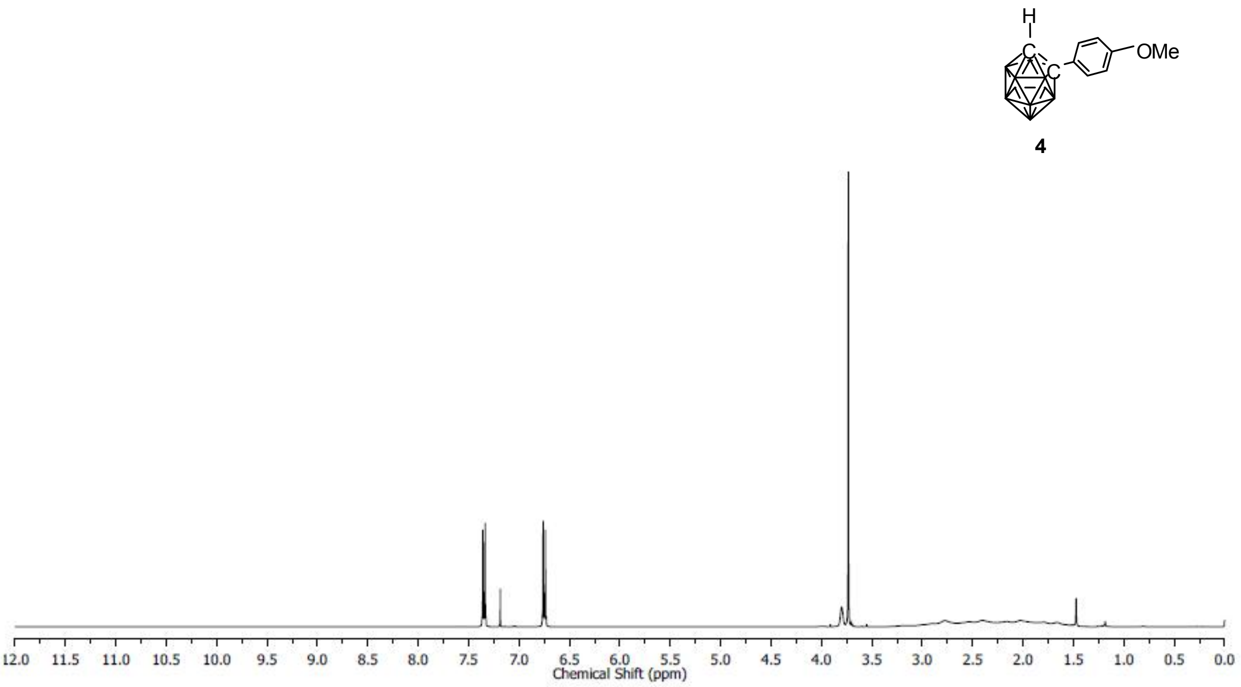

^{13}C {^{1}H}NMR of Compound 4:

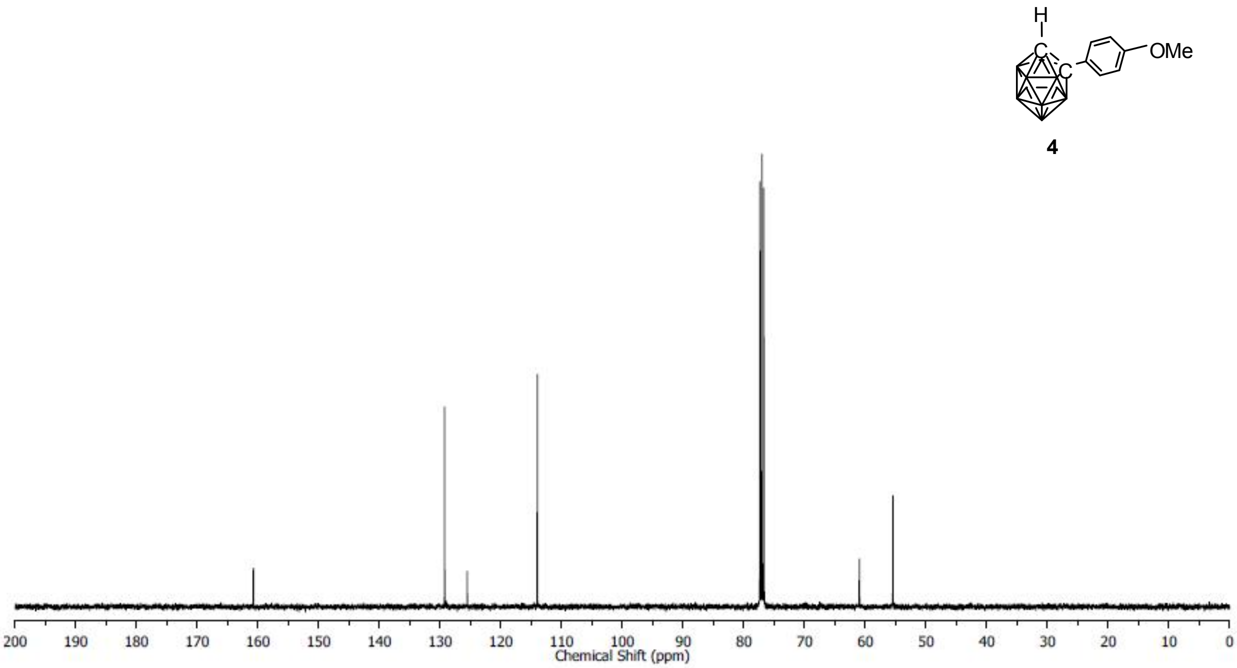

^{11}B {^{1}H}NMR of Compound 4:

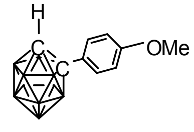

4

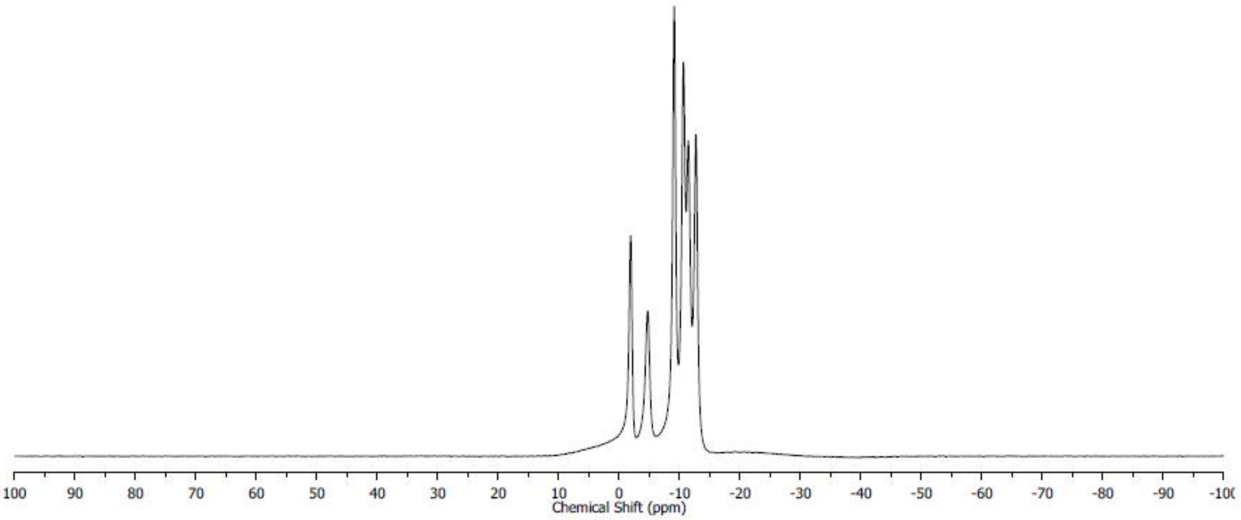

^{1}H NMR of Compound 5:

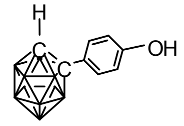

5

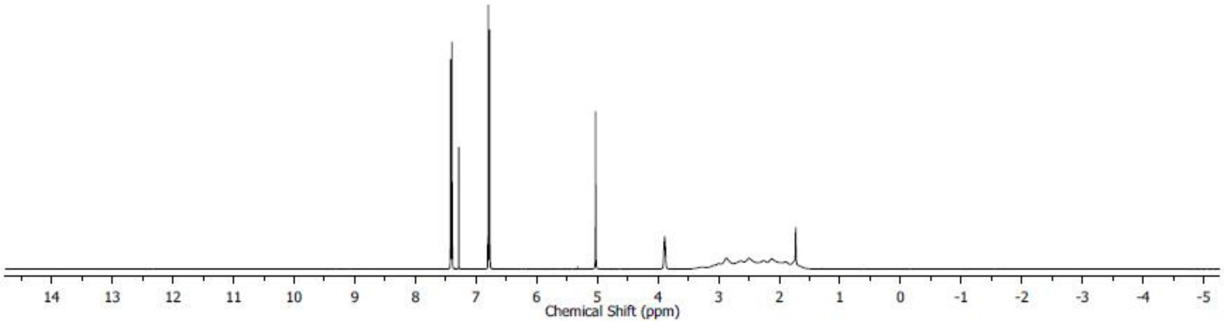

$^{13}C\ \{^{1}H\}$NMR of Compound 5:

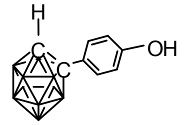

5

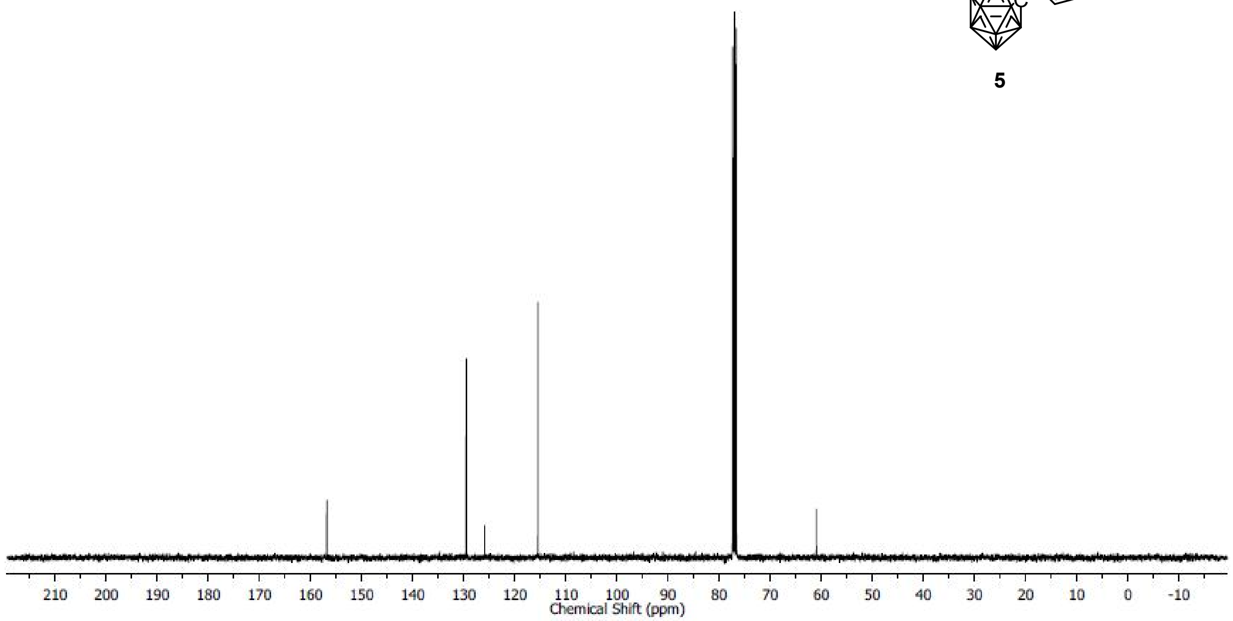

$^{11}B\ \{^{1}H\}$NMR of Compound 5:

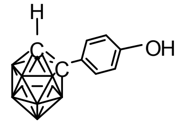

5

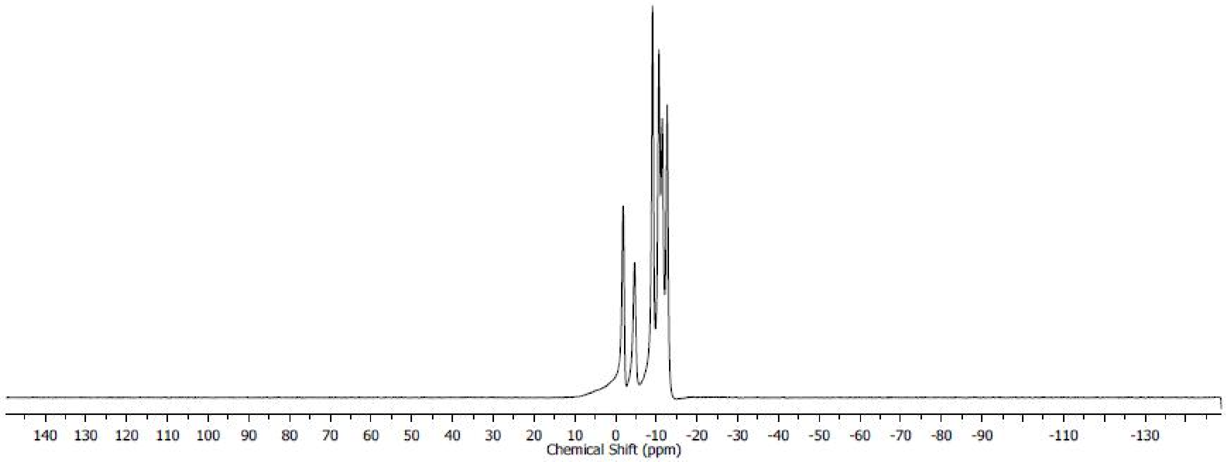

^{1}H NMR of Compound 7:

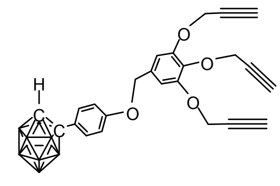

7

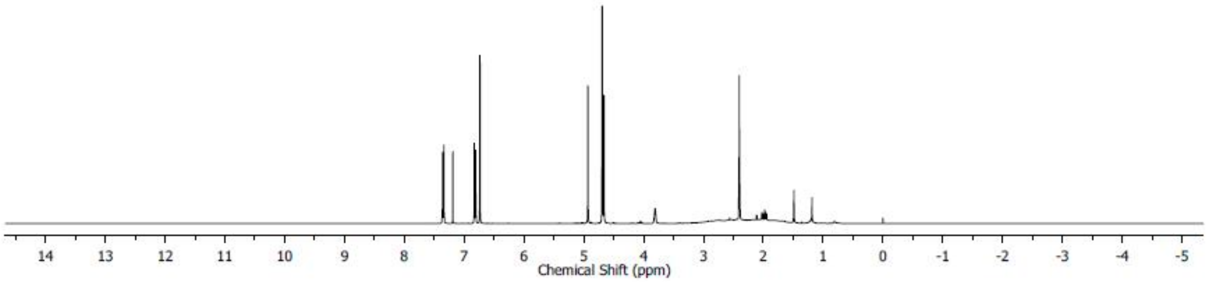

^{13}C {^{1}H} NMR of Compound 7:

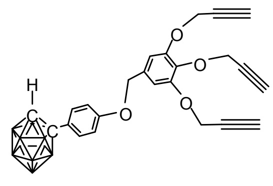

7

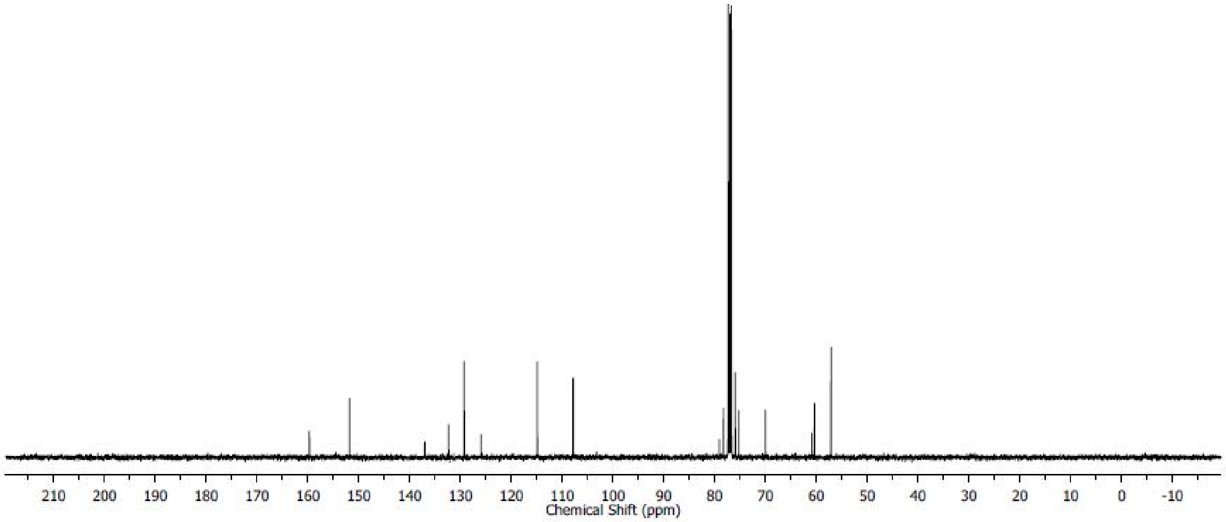

^{11}B {^{1}H}NMR of Compound 7:

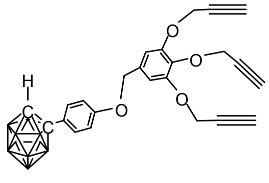

7

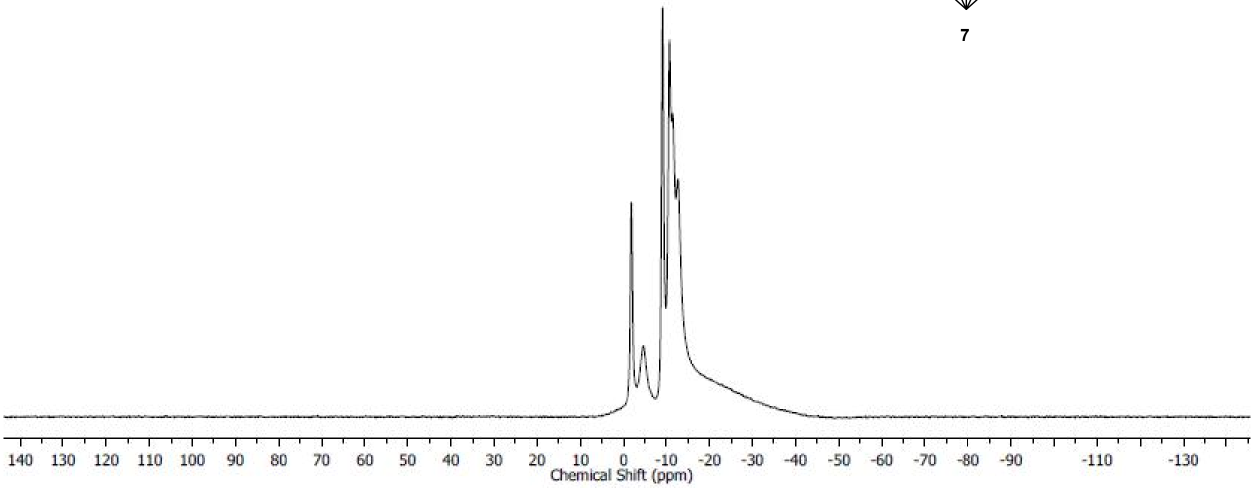

^{1}H NMR of Compound 9:

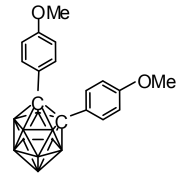

9

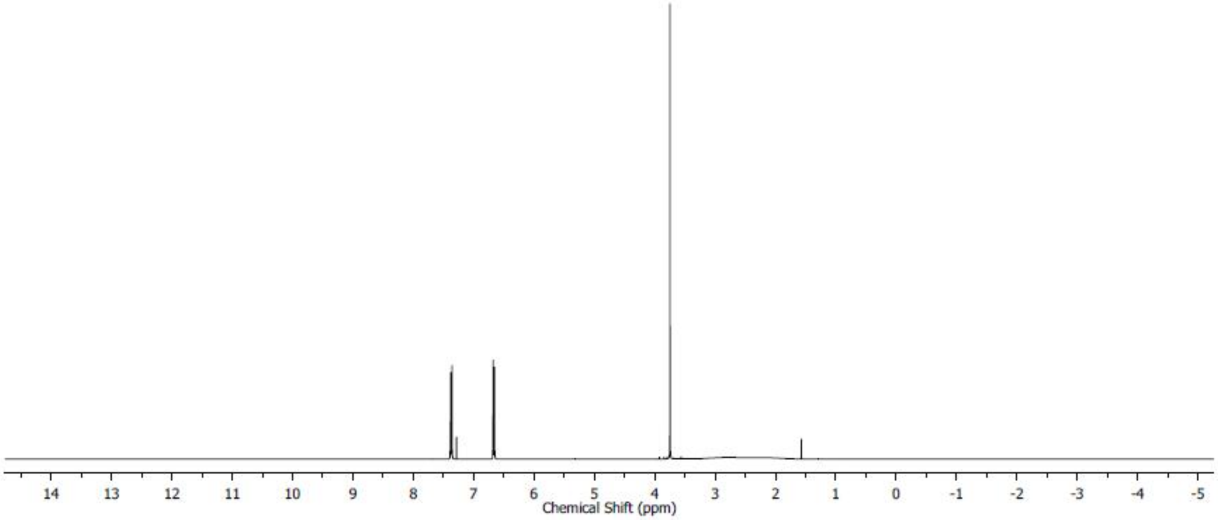

^{13}C {^{1}H} NMR of Compound 9:

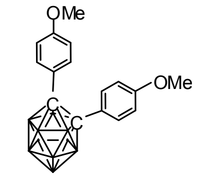

9

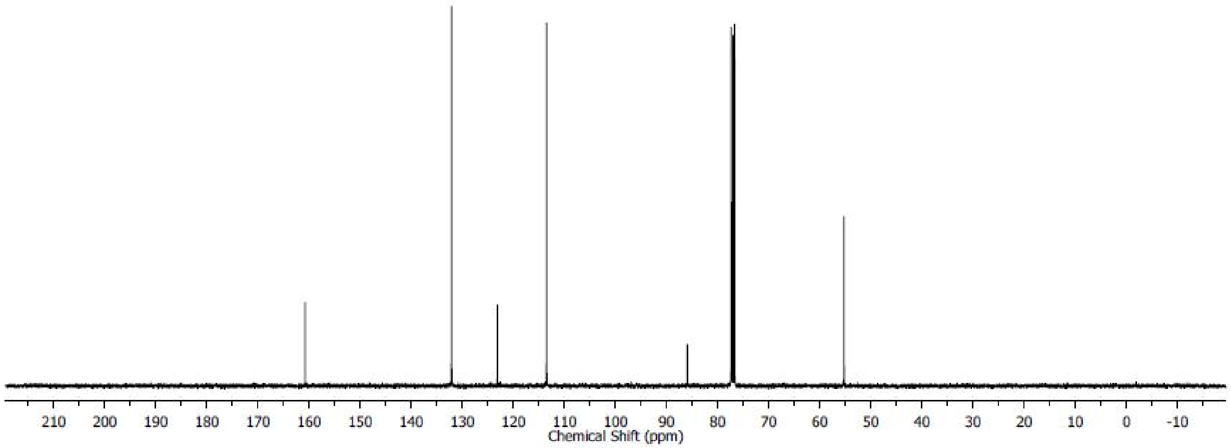

^{11}B {^{1}H}NMR of Compound 9:

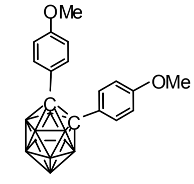

9

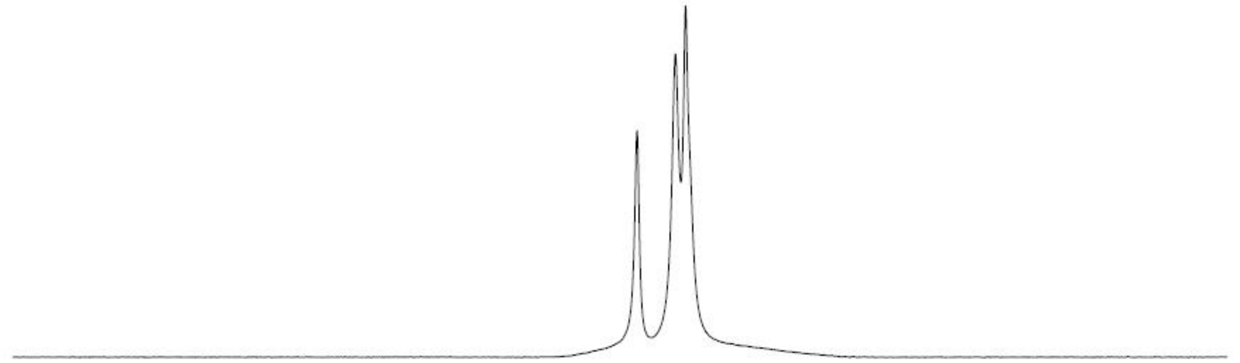

^{1}H NMR of Compound 10:

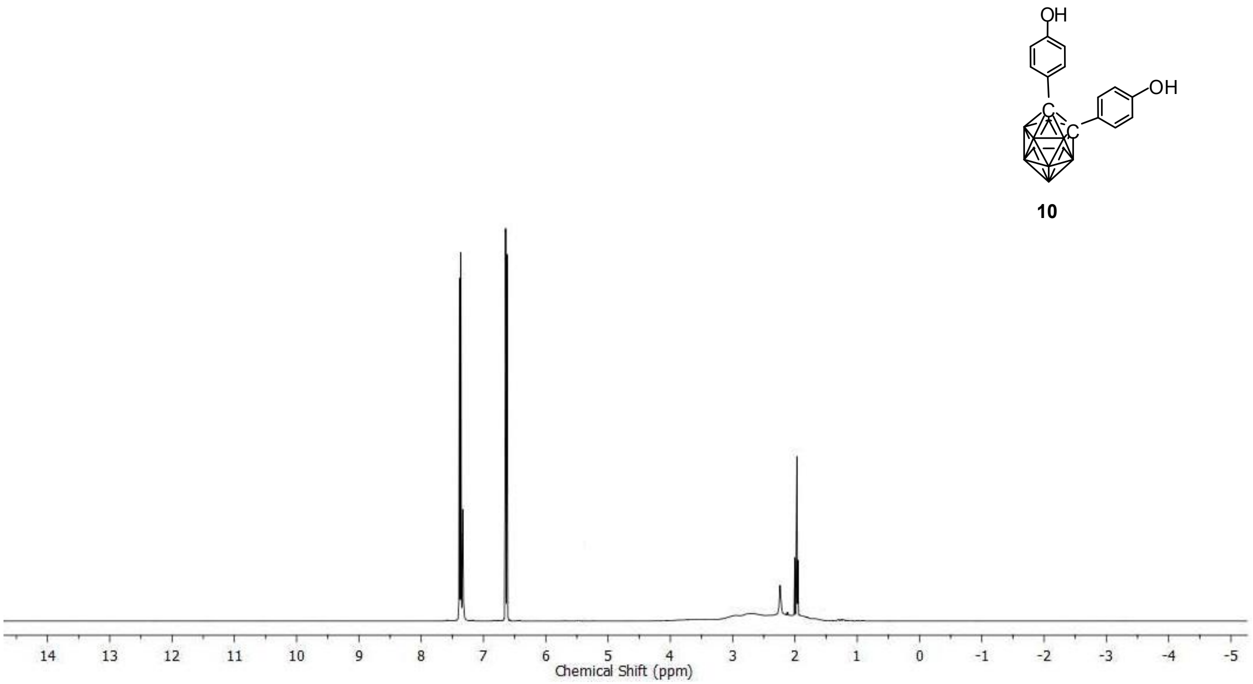

$^{13}C\{^{1}H\}$NMR of Compound 10:

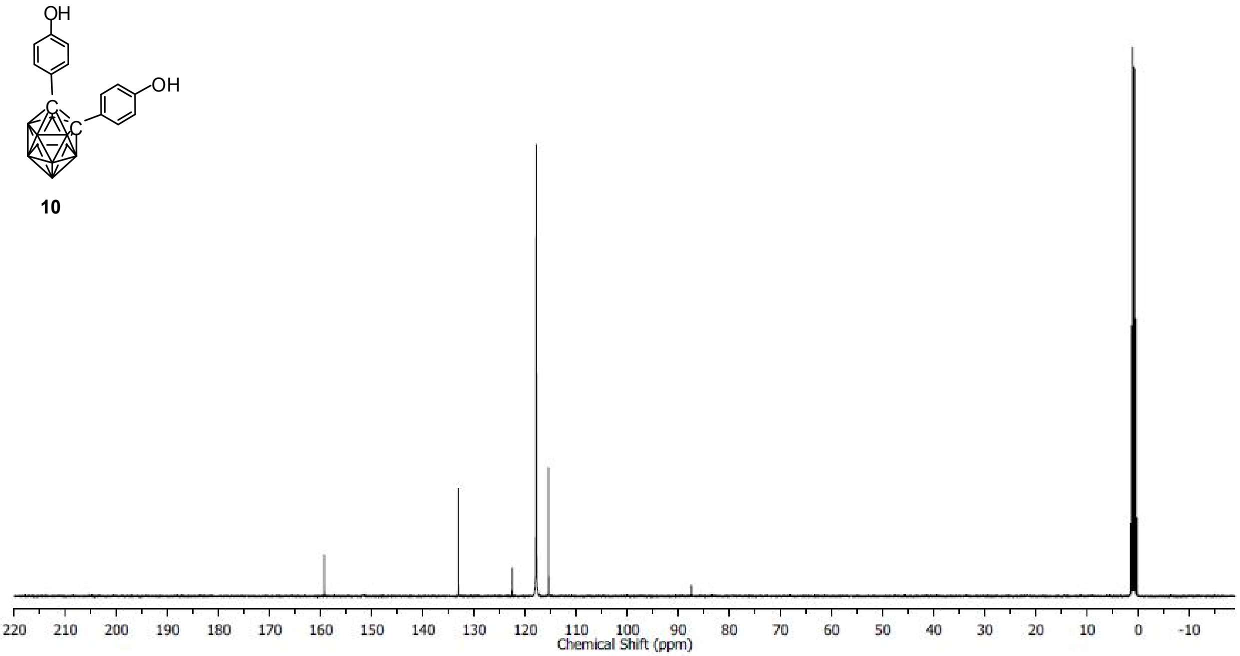

^{11}B {^{1}H}NMR of Compound 10:

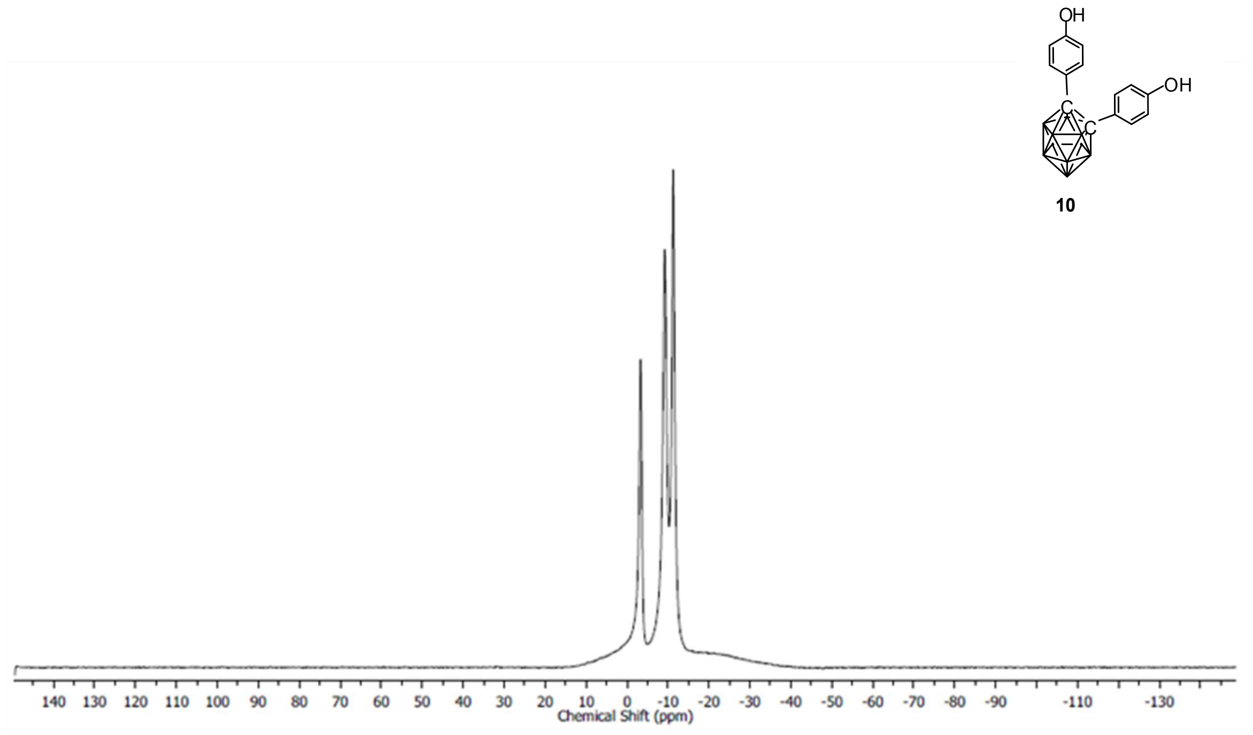

^{1}H NMR of Compound 11:

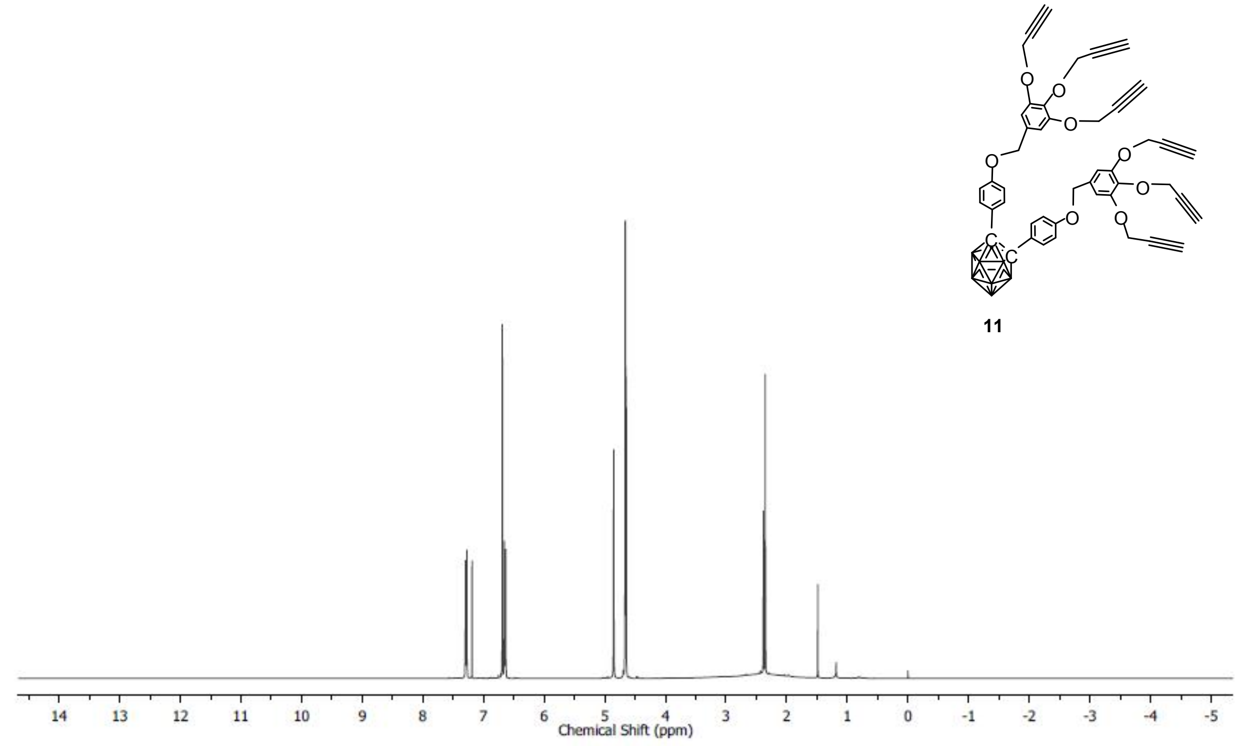

^{13}C {^{1}H}NMR of Compound 11:

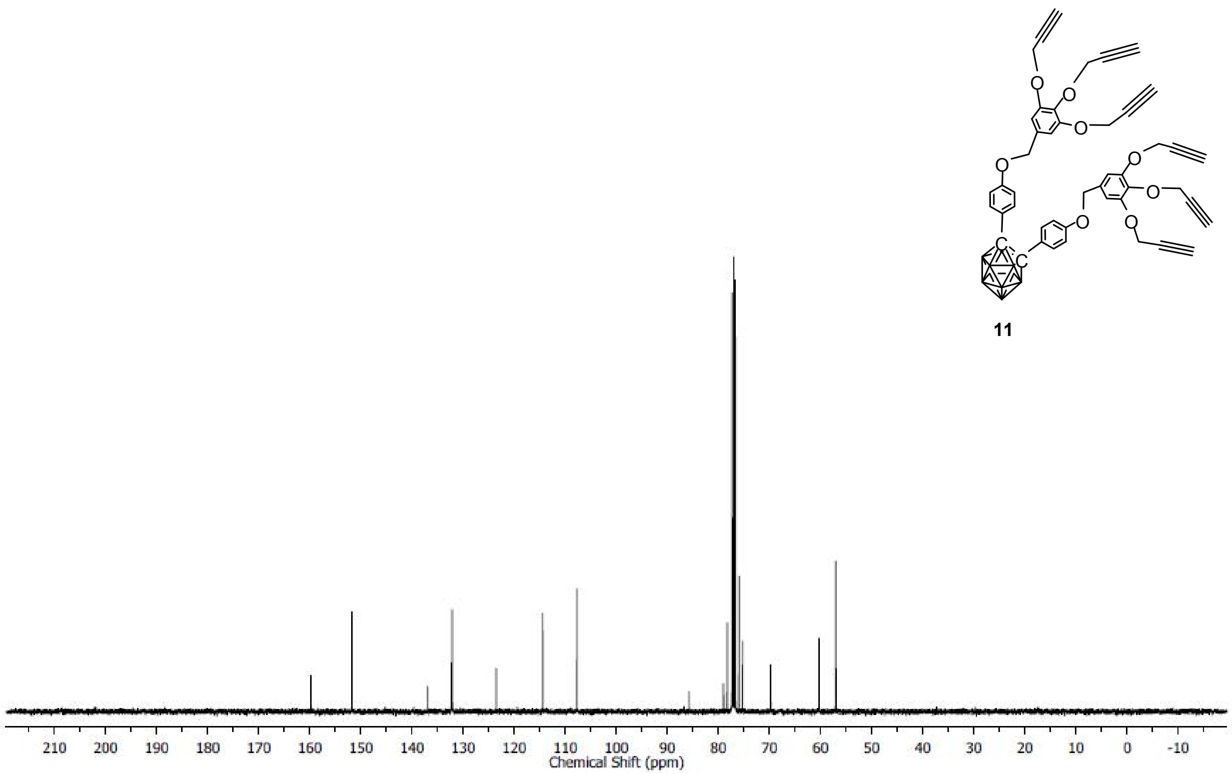

^{11}B {^{1}H}NMR of Compound 11:

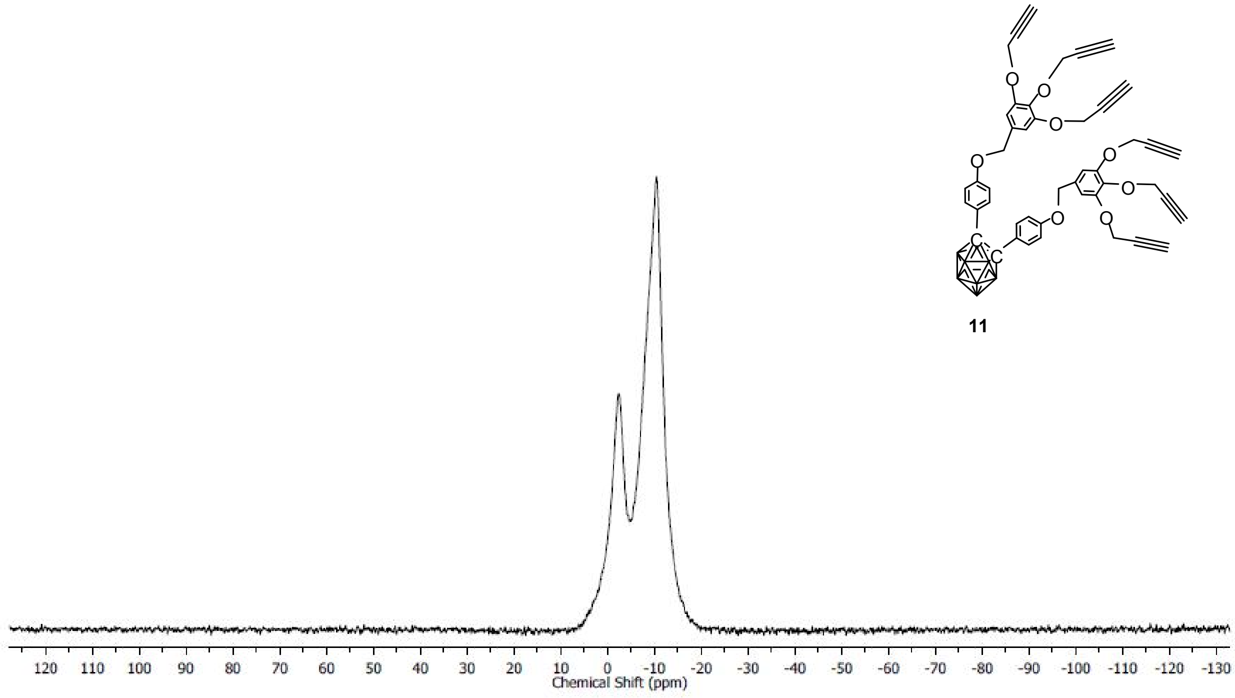

^{1}H NMR of Compound 14:

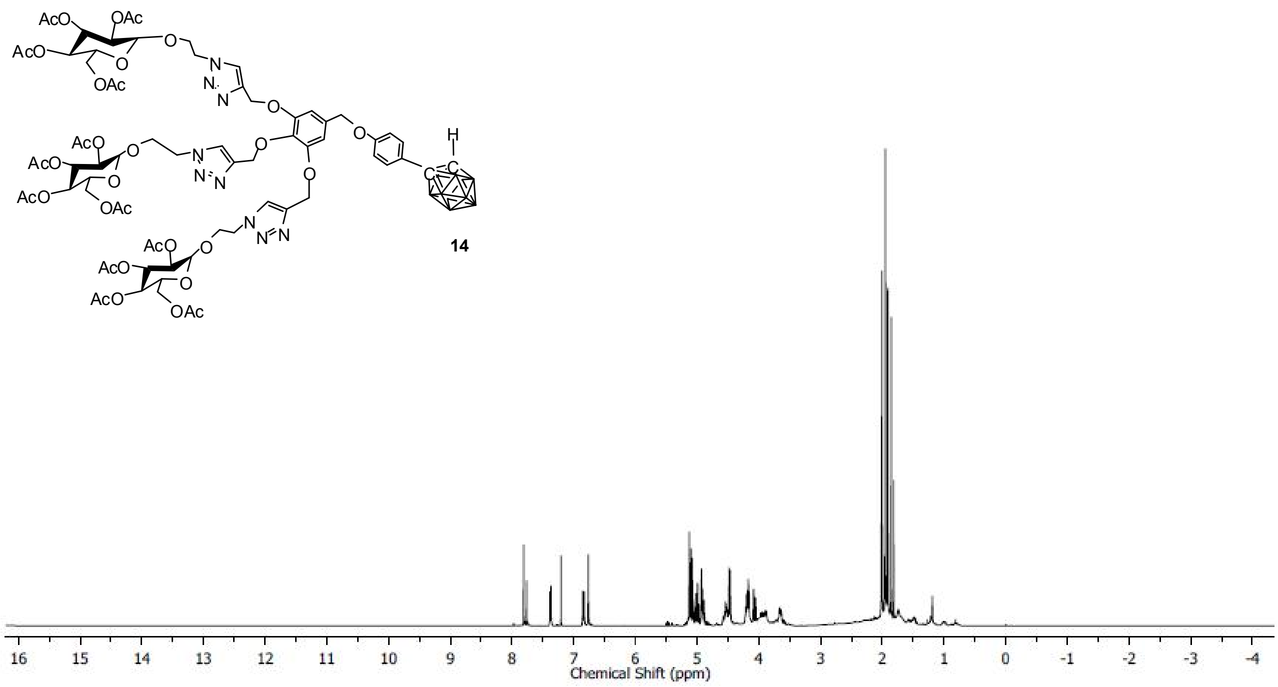

^{13}C {^{1}H}NMR of Compound 14:

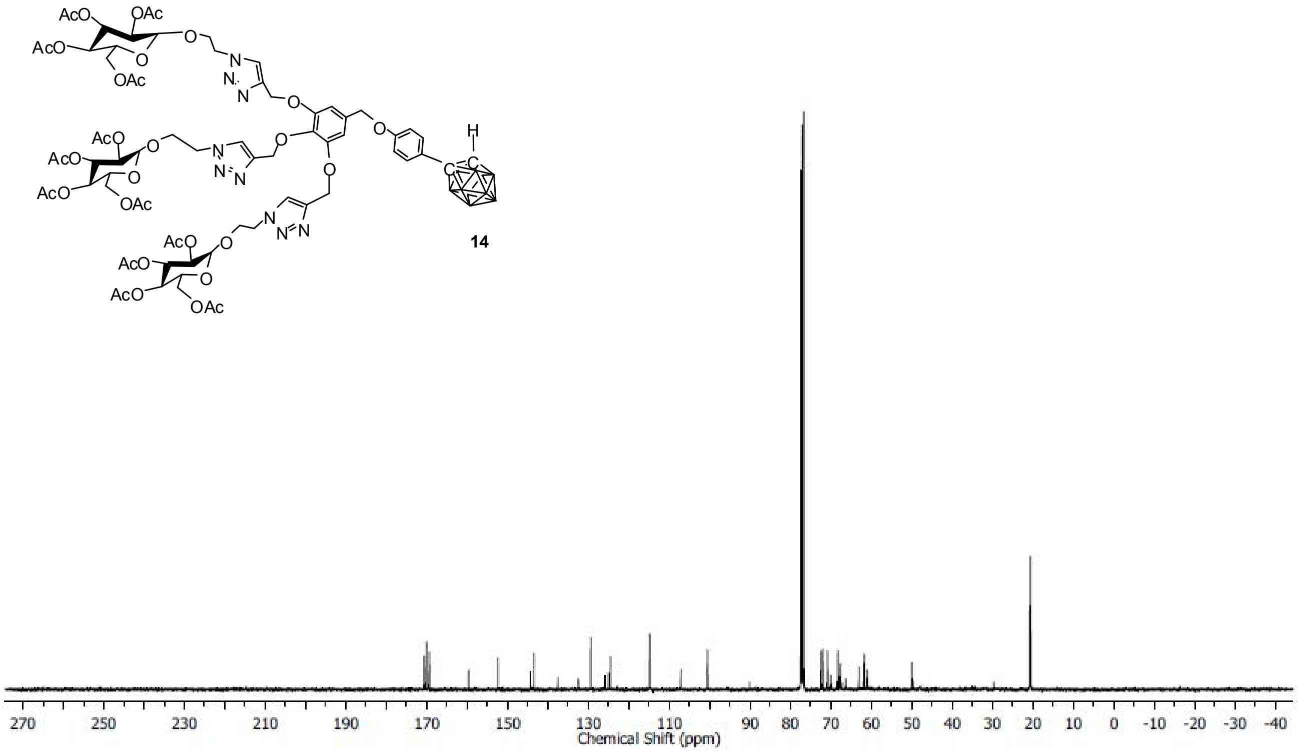

^{11}B {^{1}H}NMR of Compound 14:

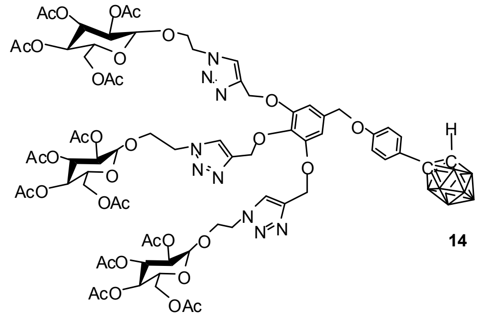

14

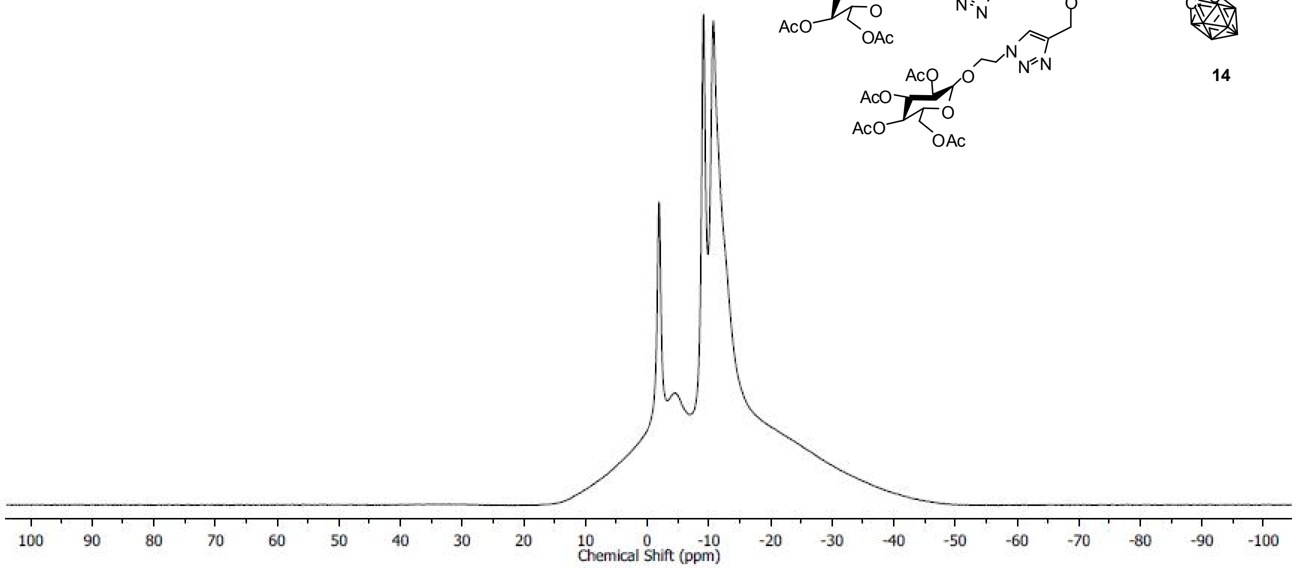

^{1}H NMR of Compound 15:

15

Glucose

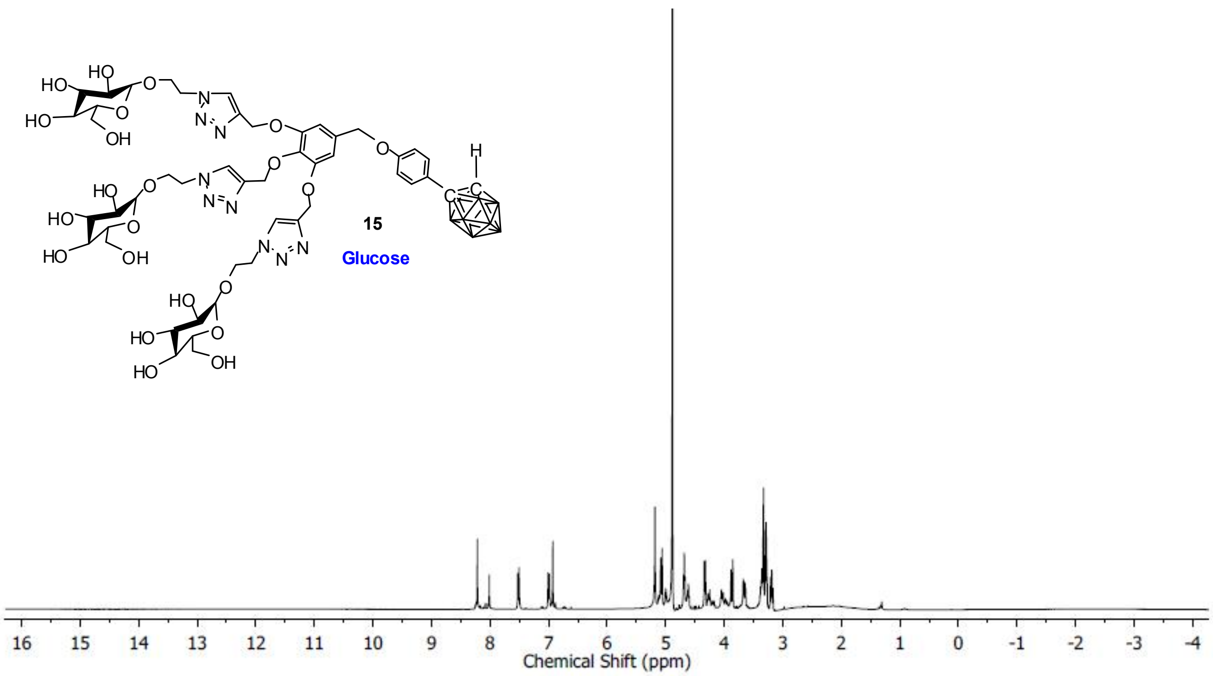

^{13}C {^{1}H}NMR of Compound 15:

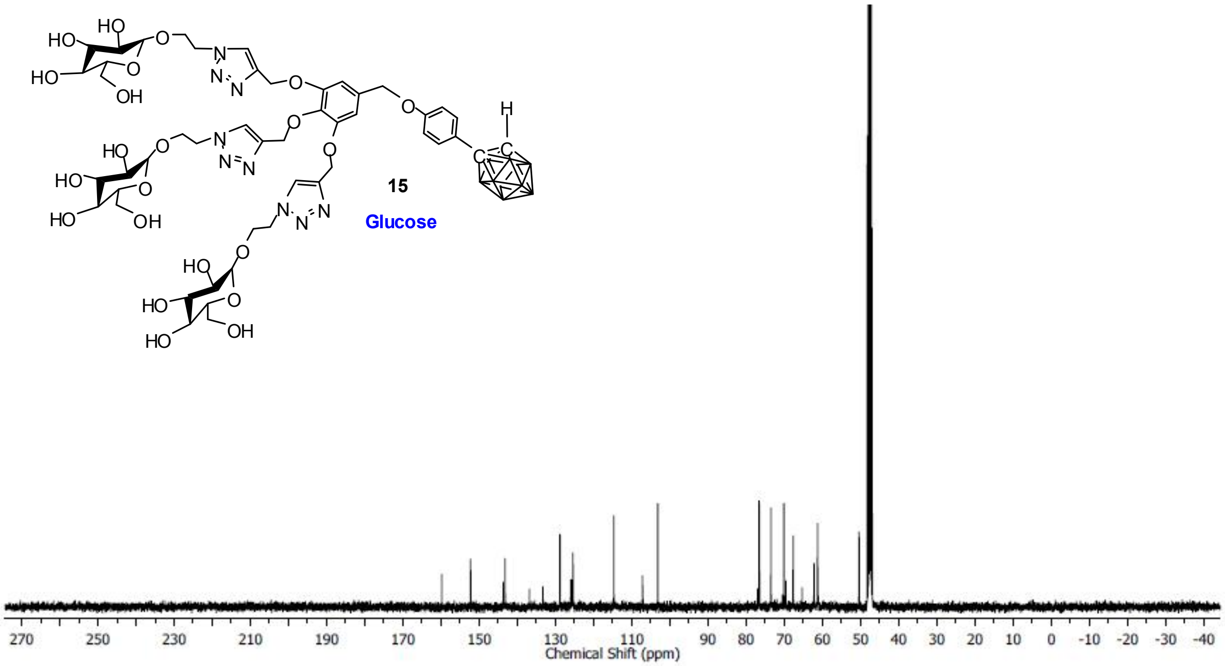

^{11}B {^{1}H}NMR of Compound 15:

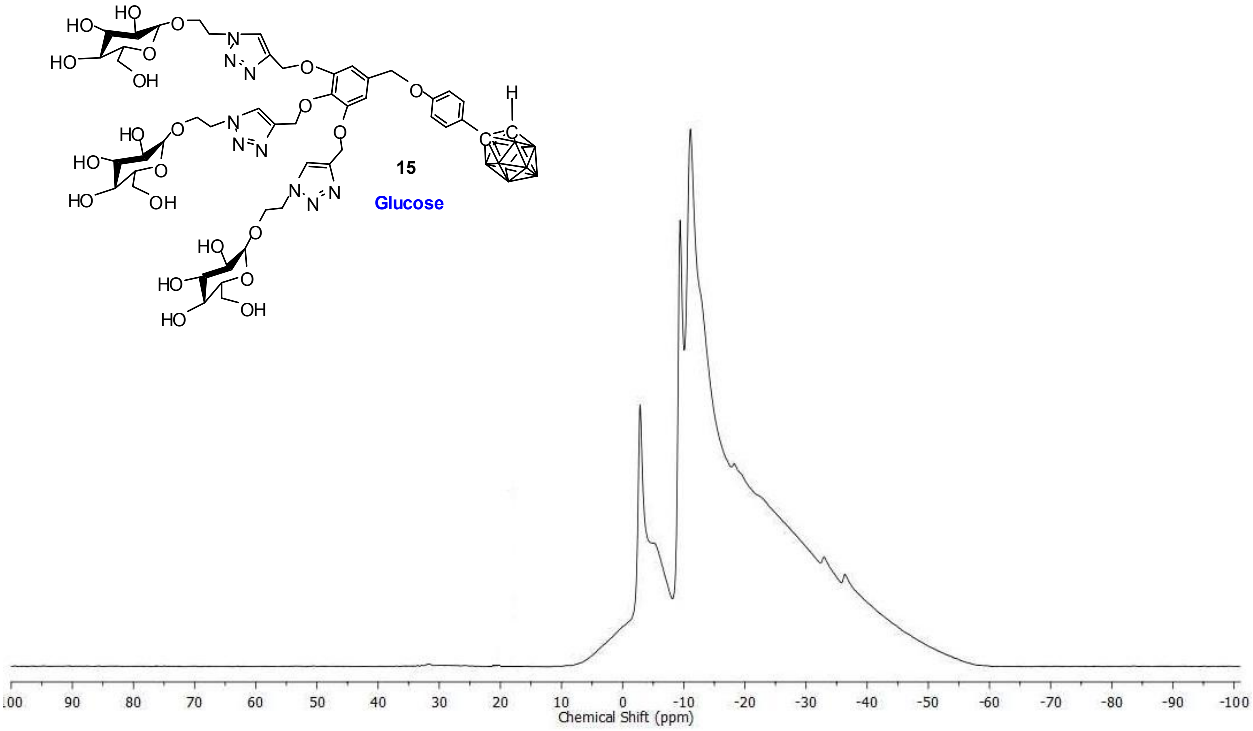

^{1}H NMR of Compound 16:

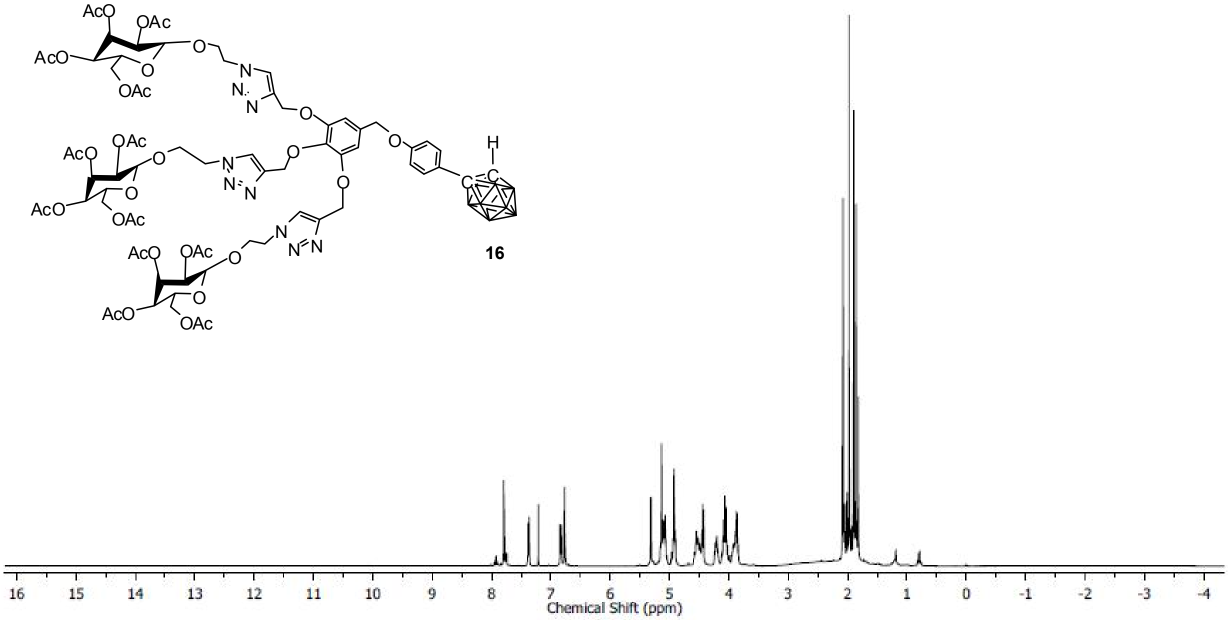

^{13}C {^{1}H}NMR of Compound 16:

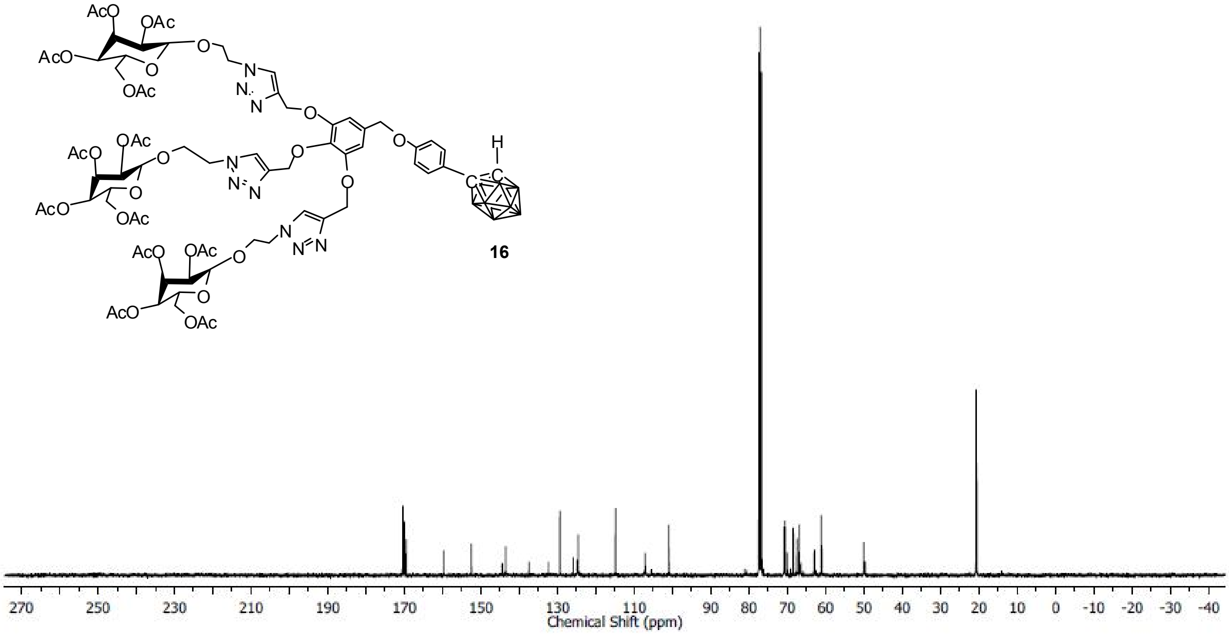

^{11}B {^{1}H} NMR of Compound 16:

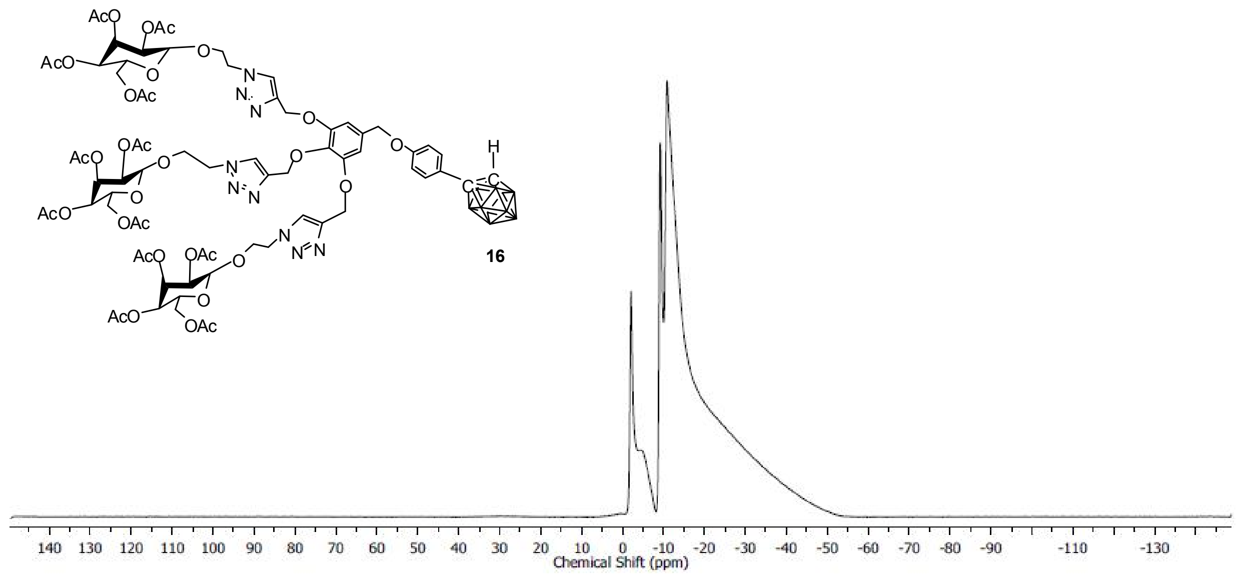

^{1}H NMR of Compound 17:

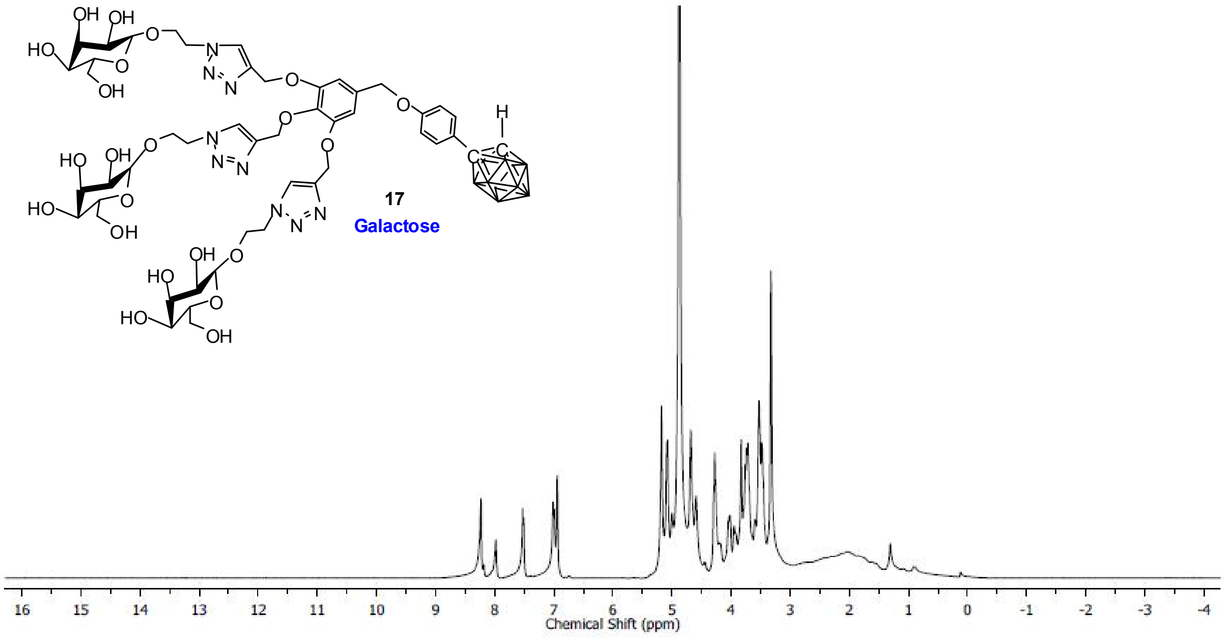

^{13}C {^{1}H}NMR of Compound 17:

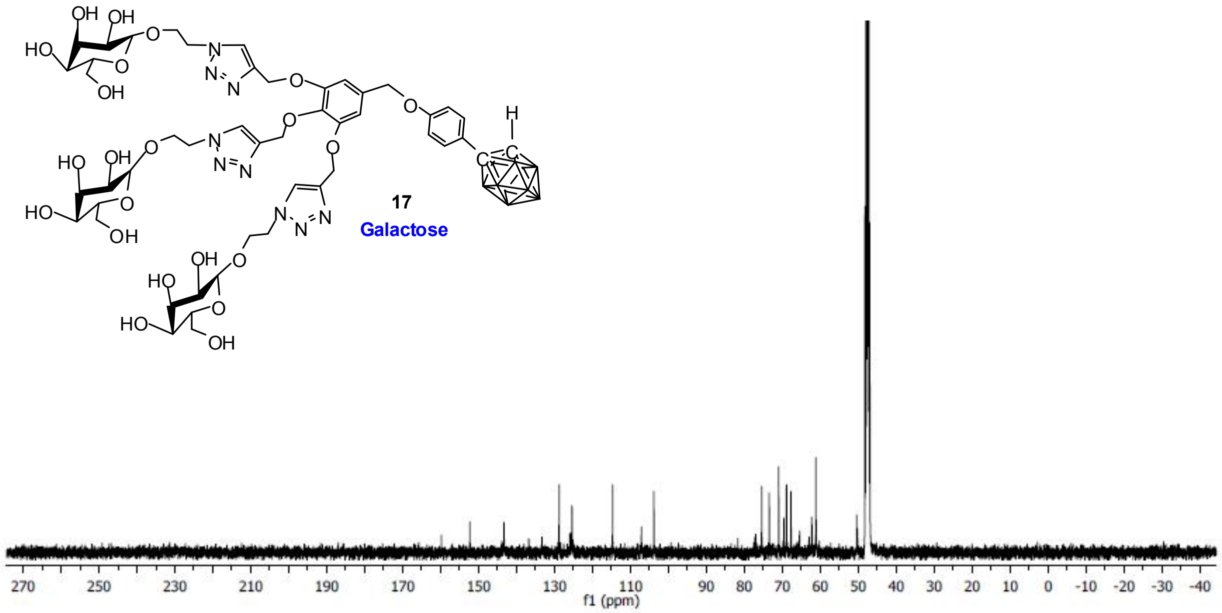

^{11}B {^{1}H} NMR of Compound 17:

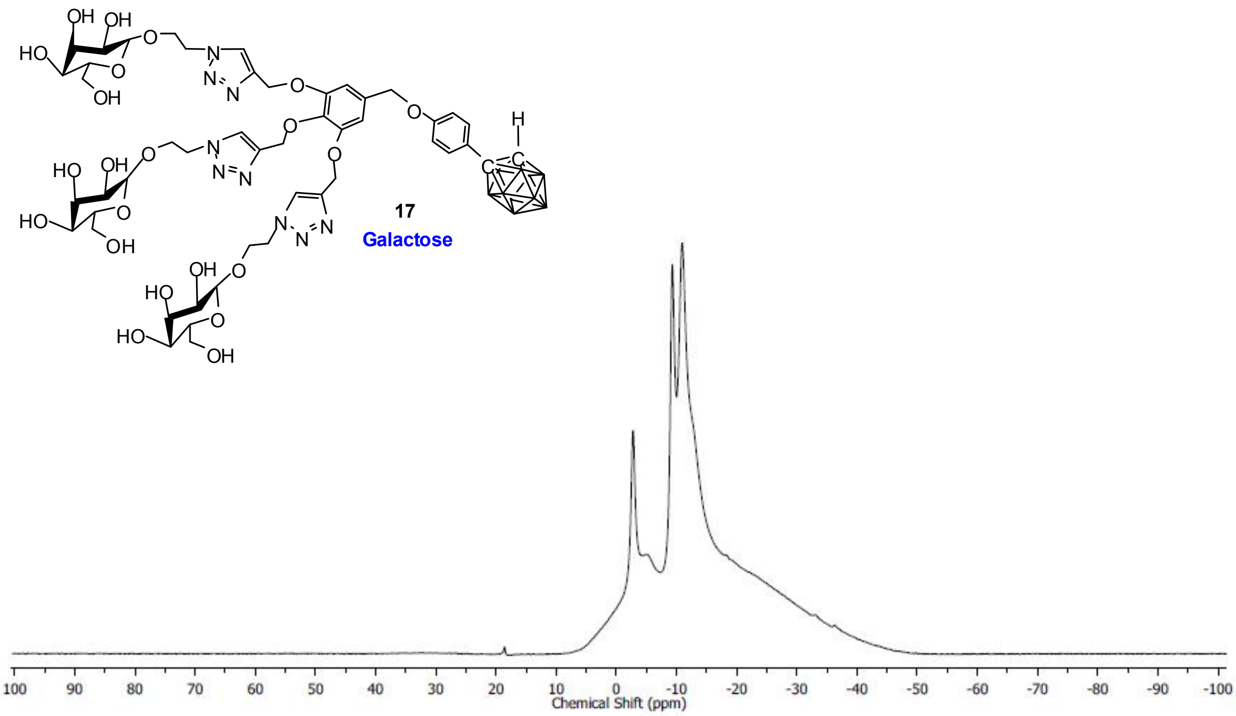

^{1}H NMR of Compound 18:

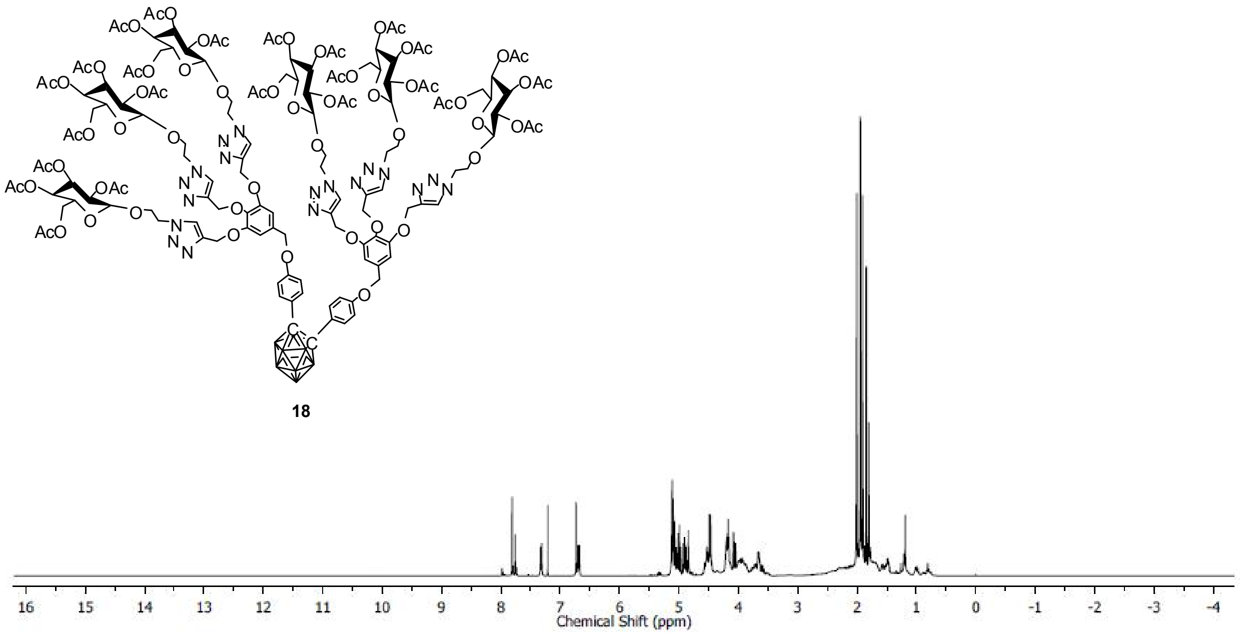

18

^{13}C {^{1}H}NMR of Compound 18:

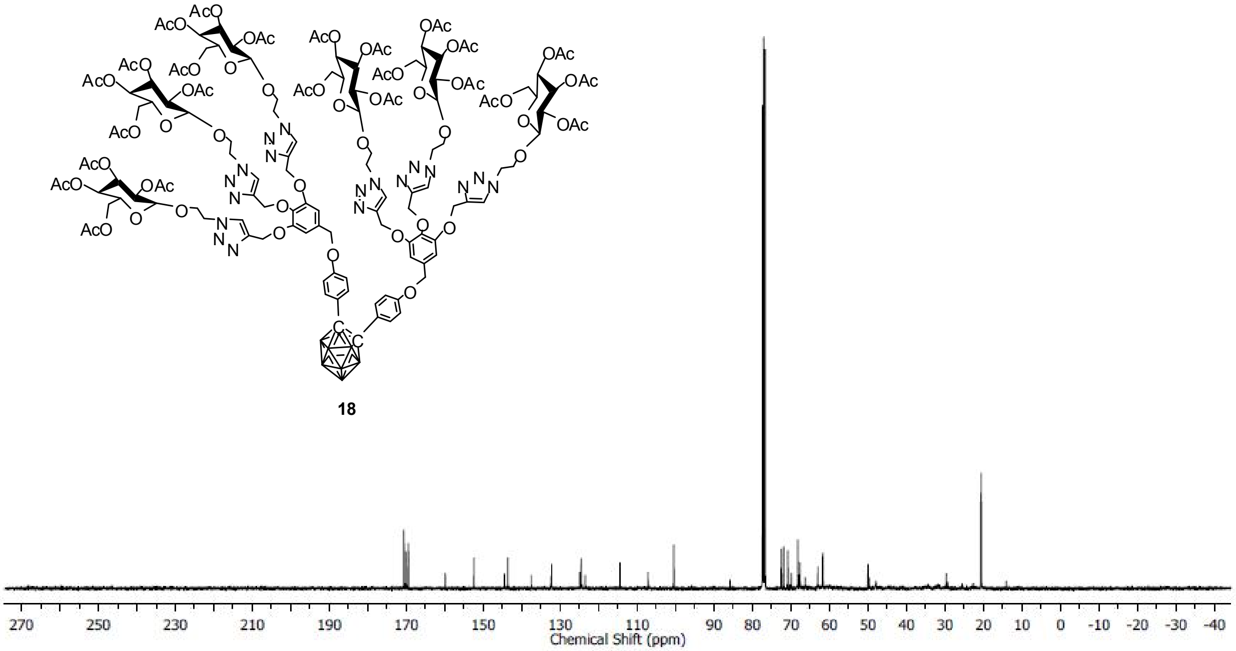

18

^{11}B {^{1}H}NMR of Compound 18:

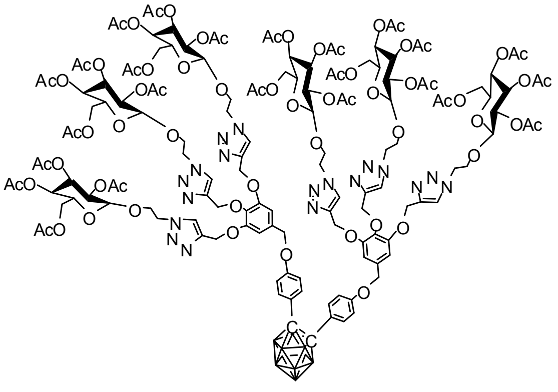

18

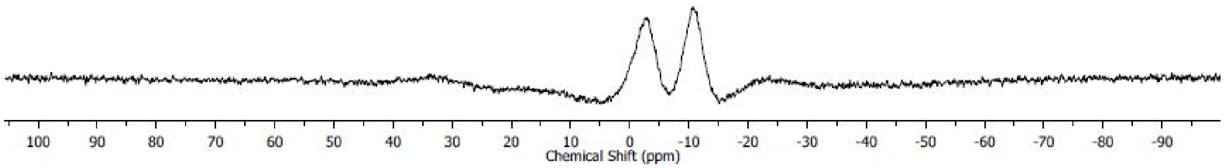

^{1}H NMR of Compound 19:

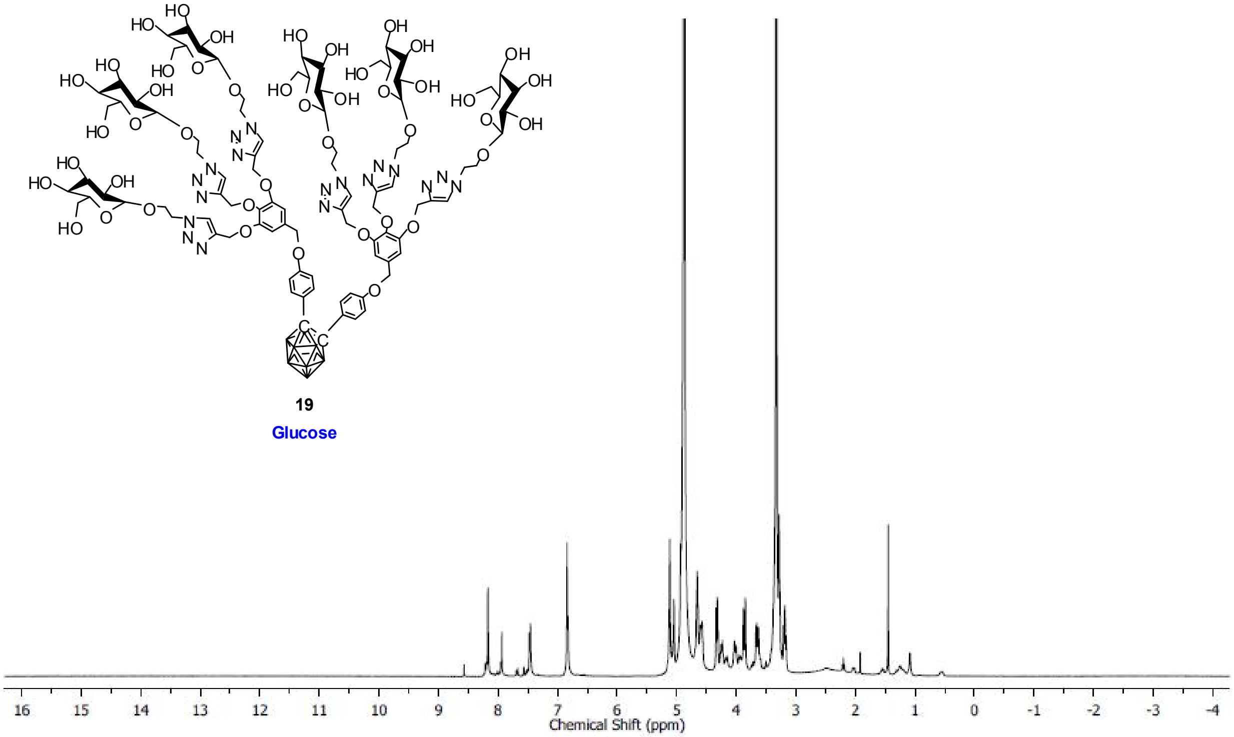

19

Glucose

^{13}C {^{1}H}NMR of Compound 19:

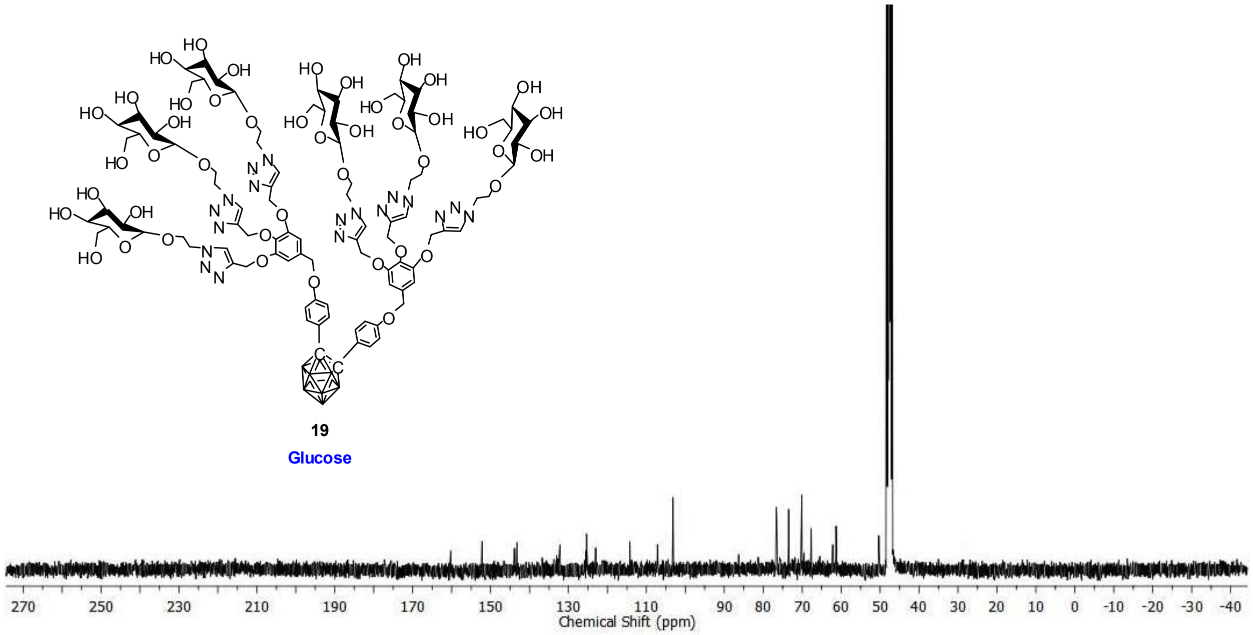

^{11}B {^{1}H}NMR of Compound 19:

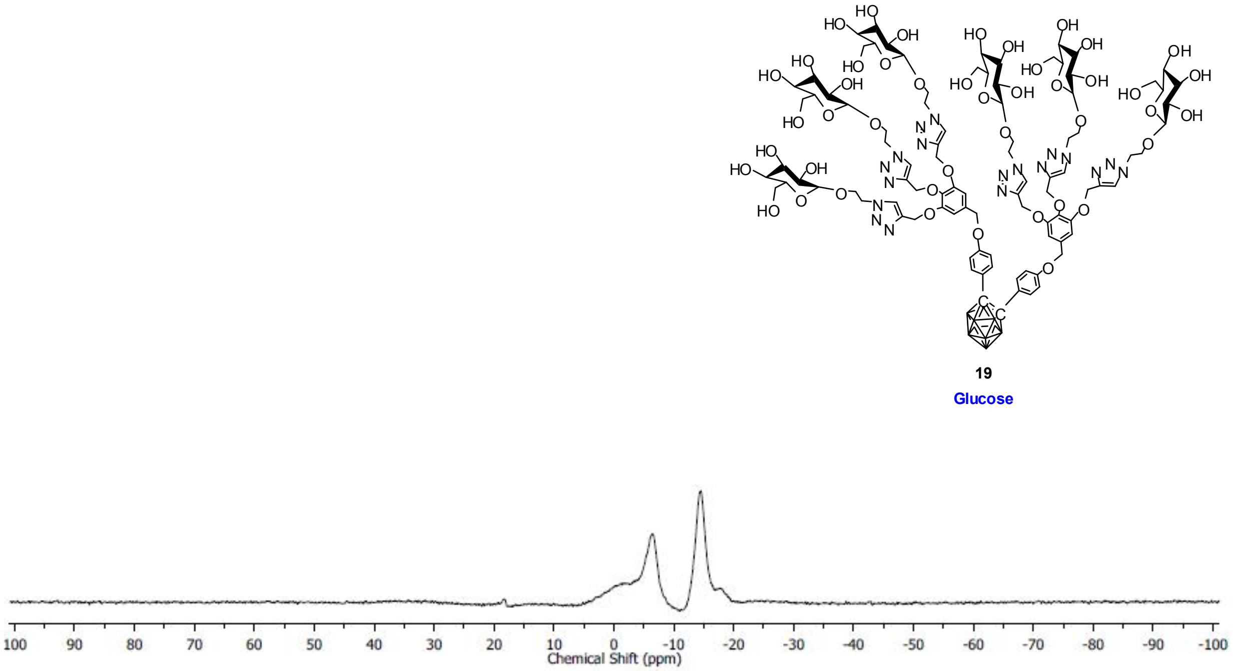

^{1}H NMR of Compound 20:

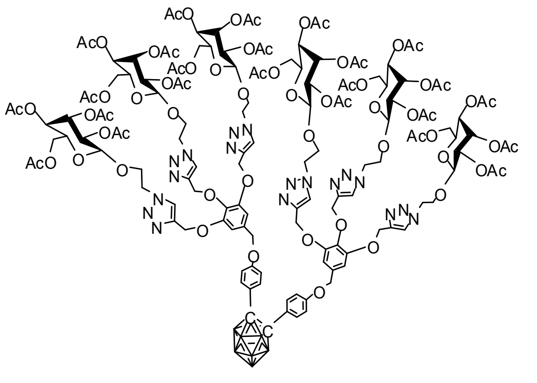

20

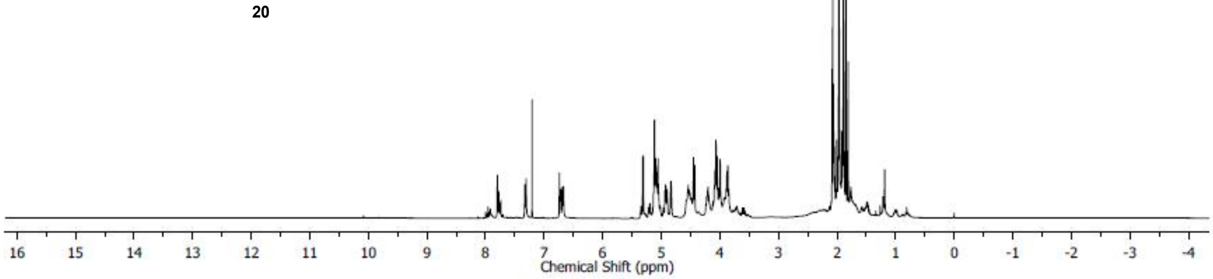

^{13}C {^{1}H}NMR of Compound 20:

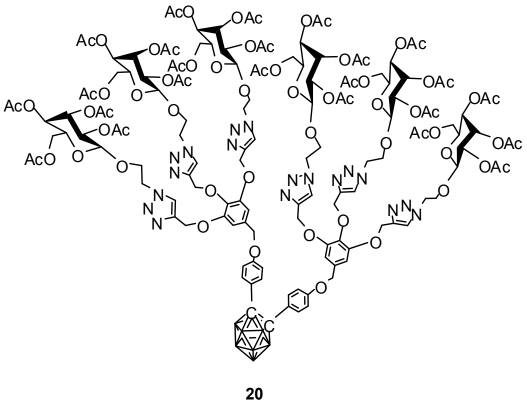

20

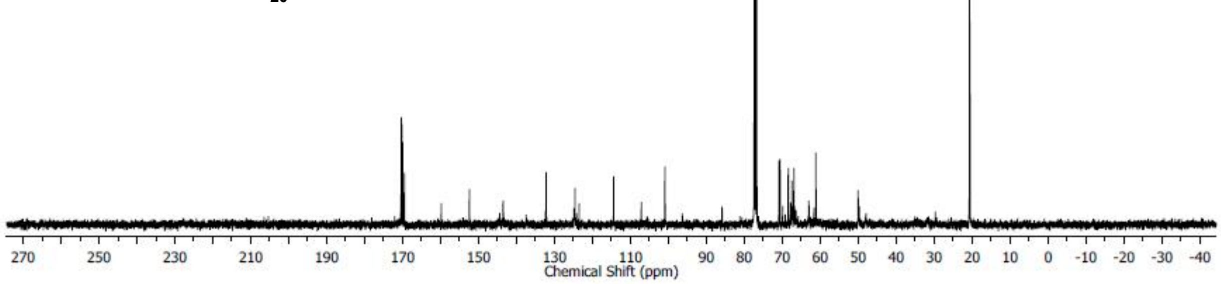

^{11}B {^{1}H}NMR of Compound 20:

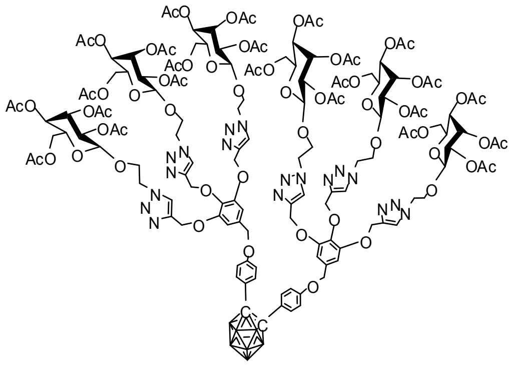

20

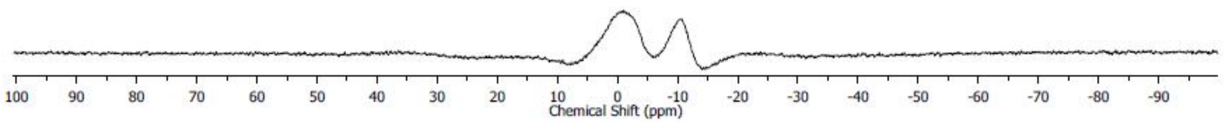

^{1}H NMR of Compound 21:

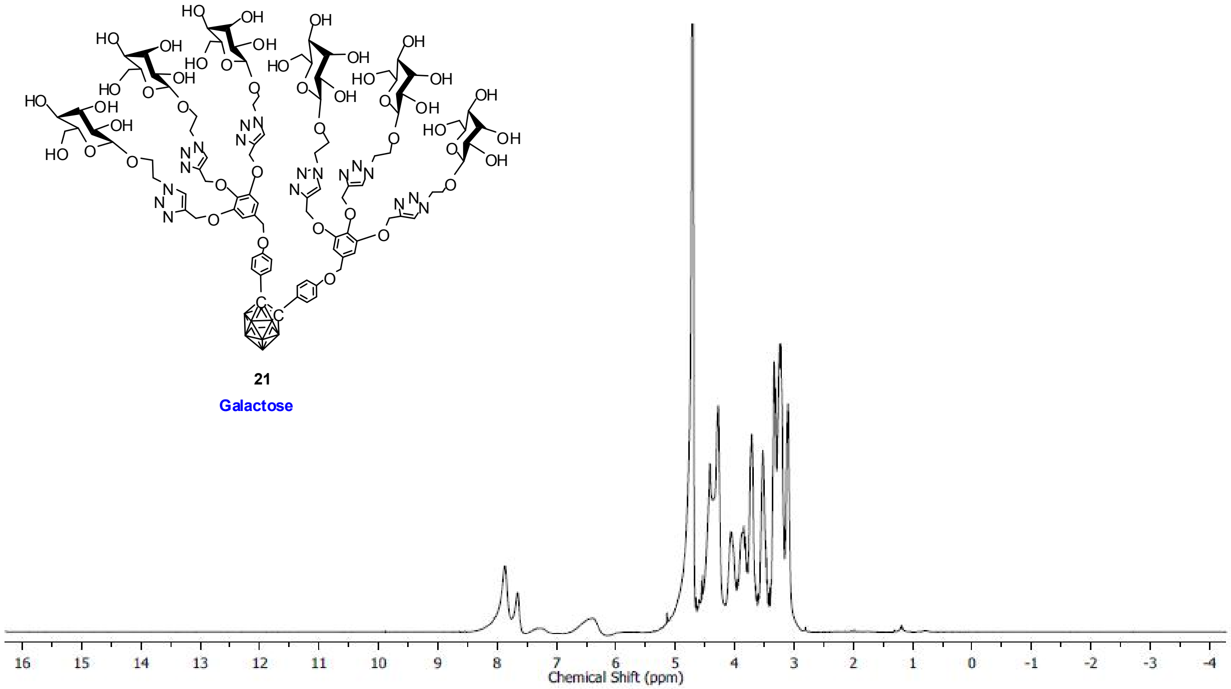

21

Galactose

^{13}C {^{1}H}NMR of Compound 21:

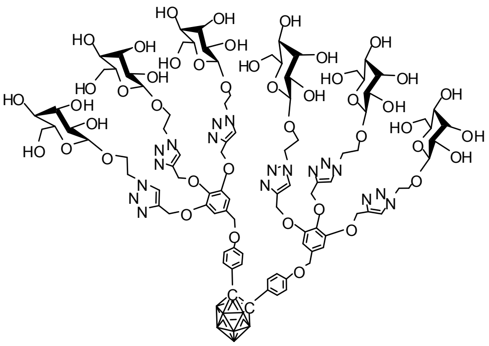

21

Galactose

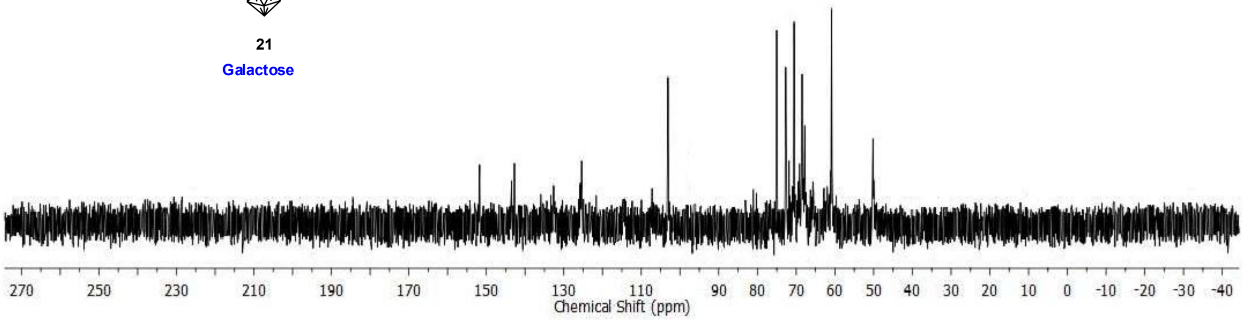

^{11}B {^{1}H}NMR of Compound 21:

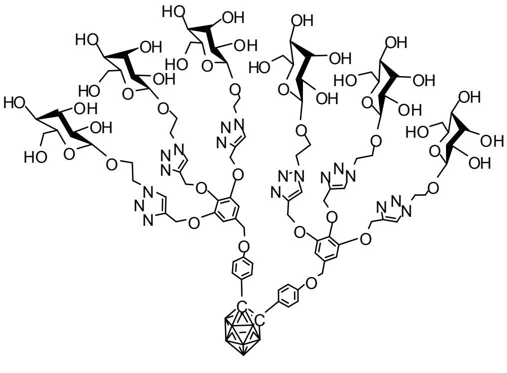

21

Galactose

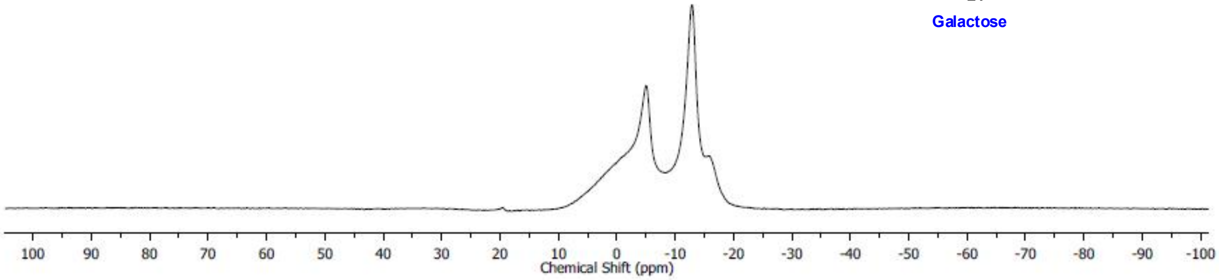

MASS Spectra

Compound 4:

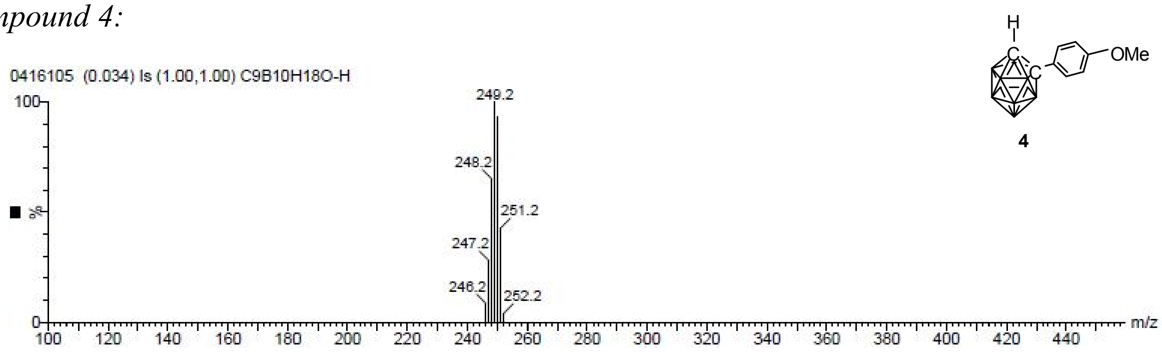

4

Compound 5:

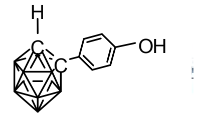

5

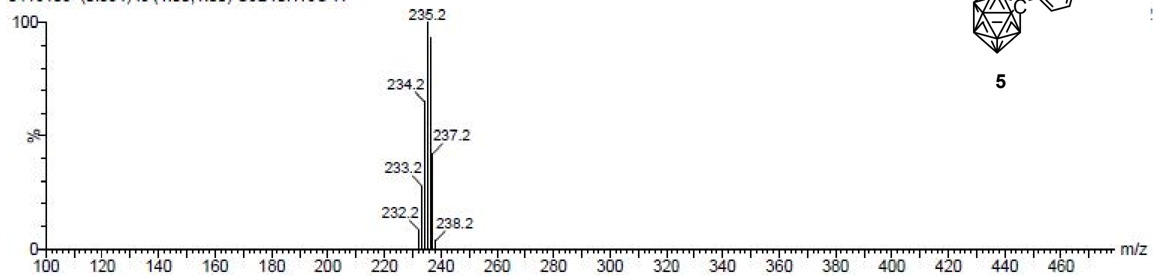

Compound 7:

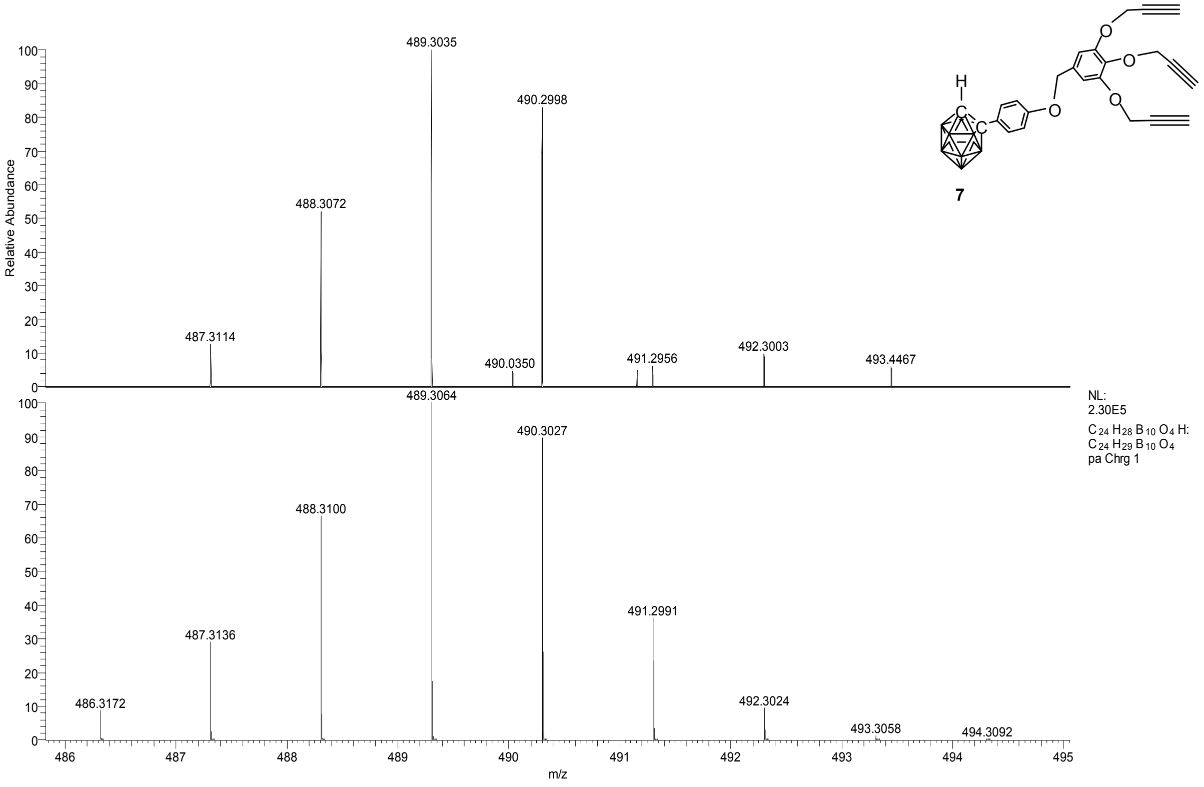

7

Compound 9:

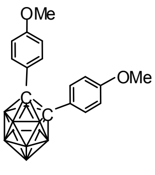

9

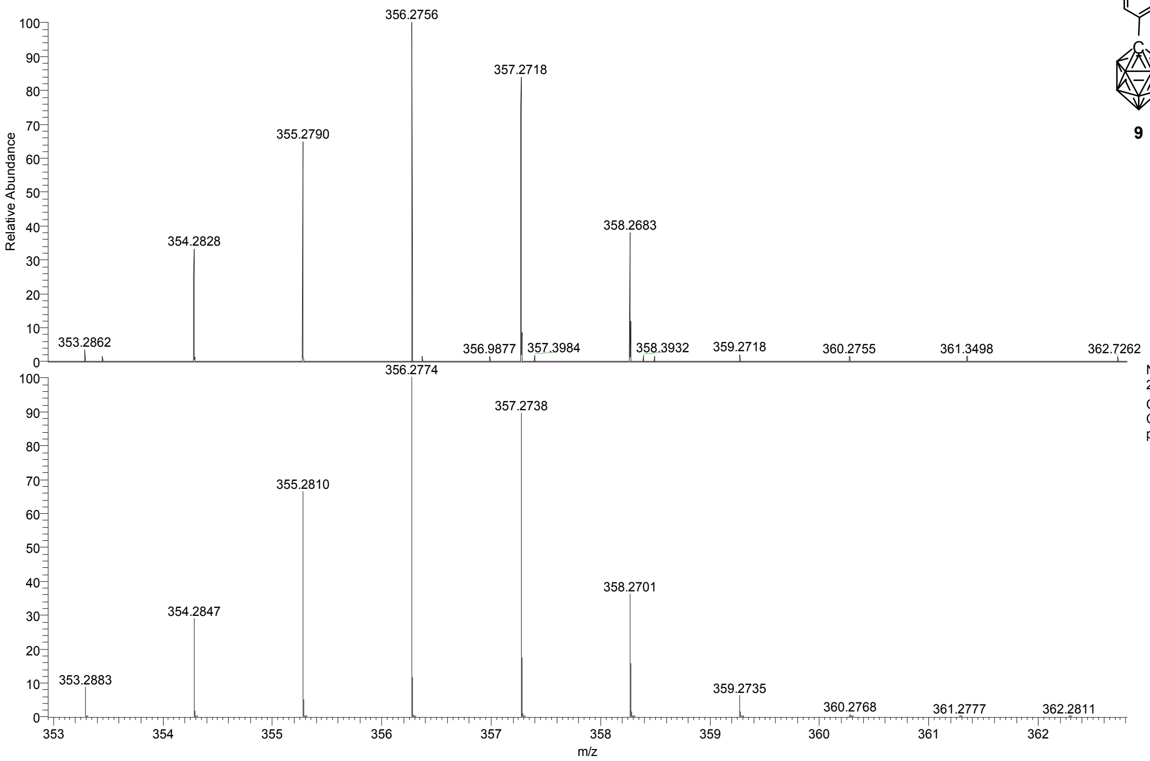

Compound 10:

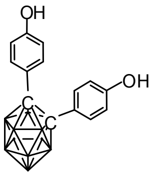

10

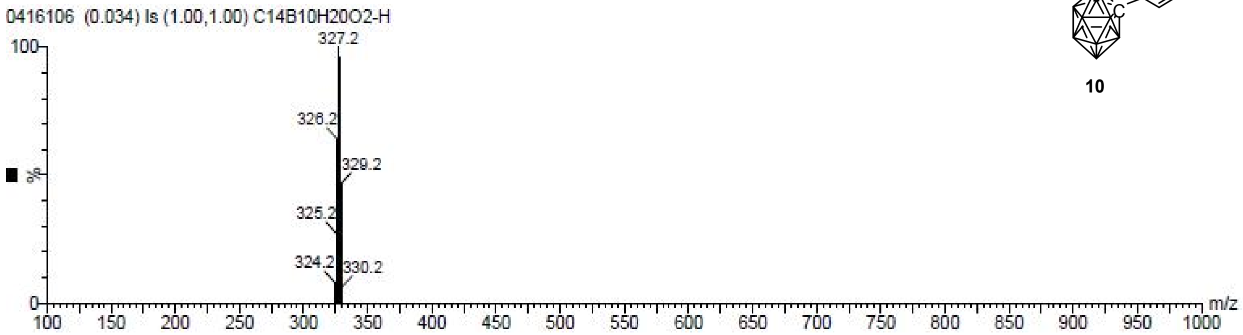

Compound 11:

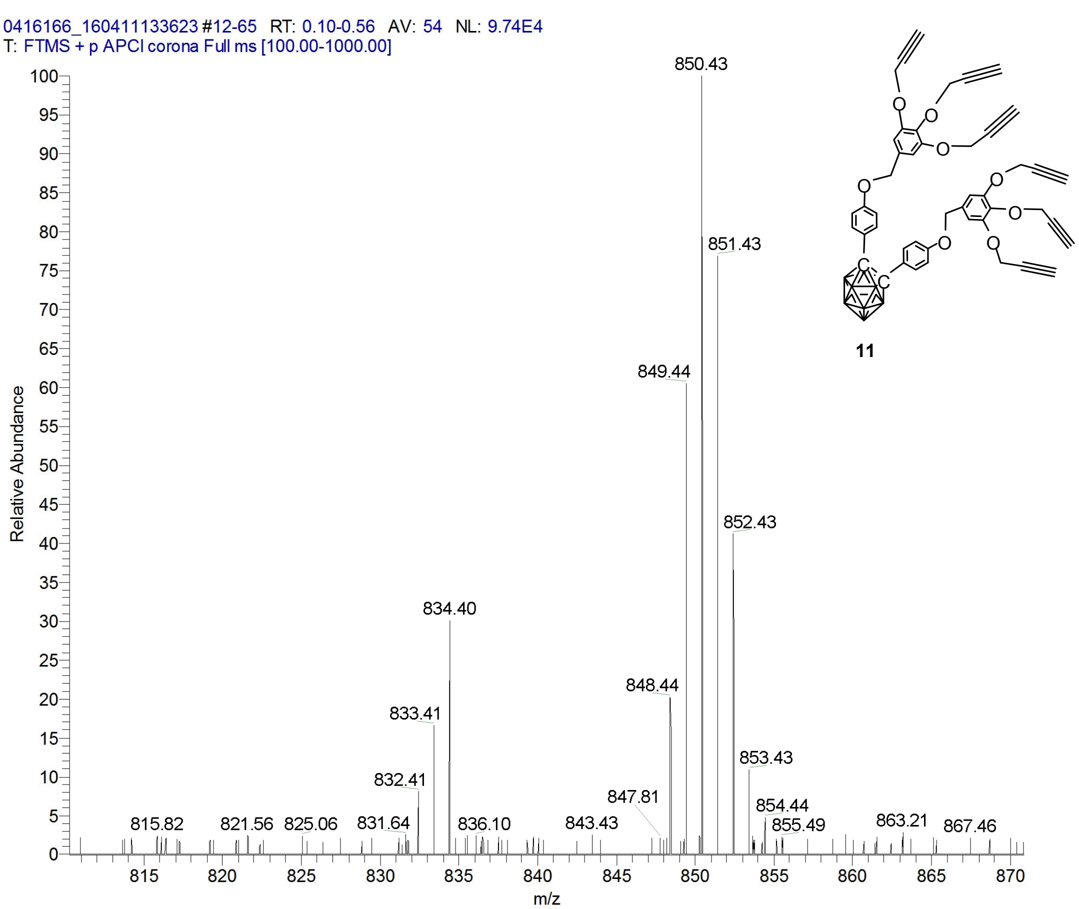

Compound 14:

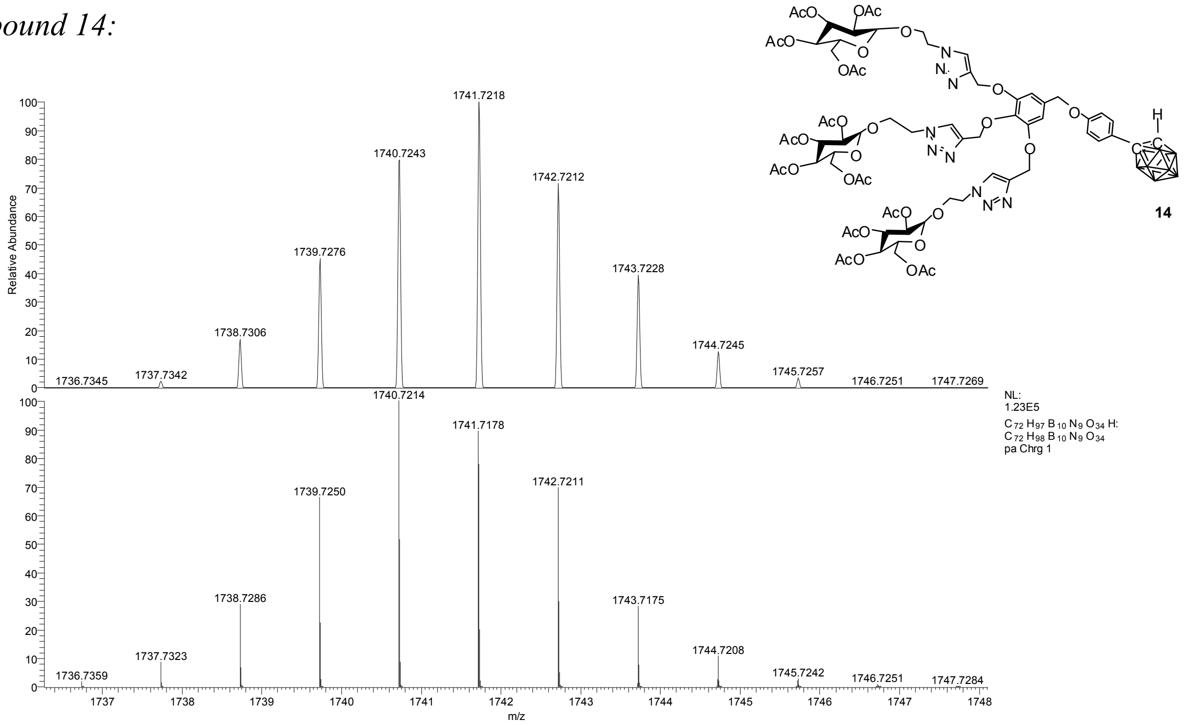

Compound 15:

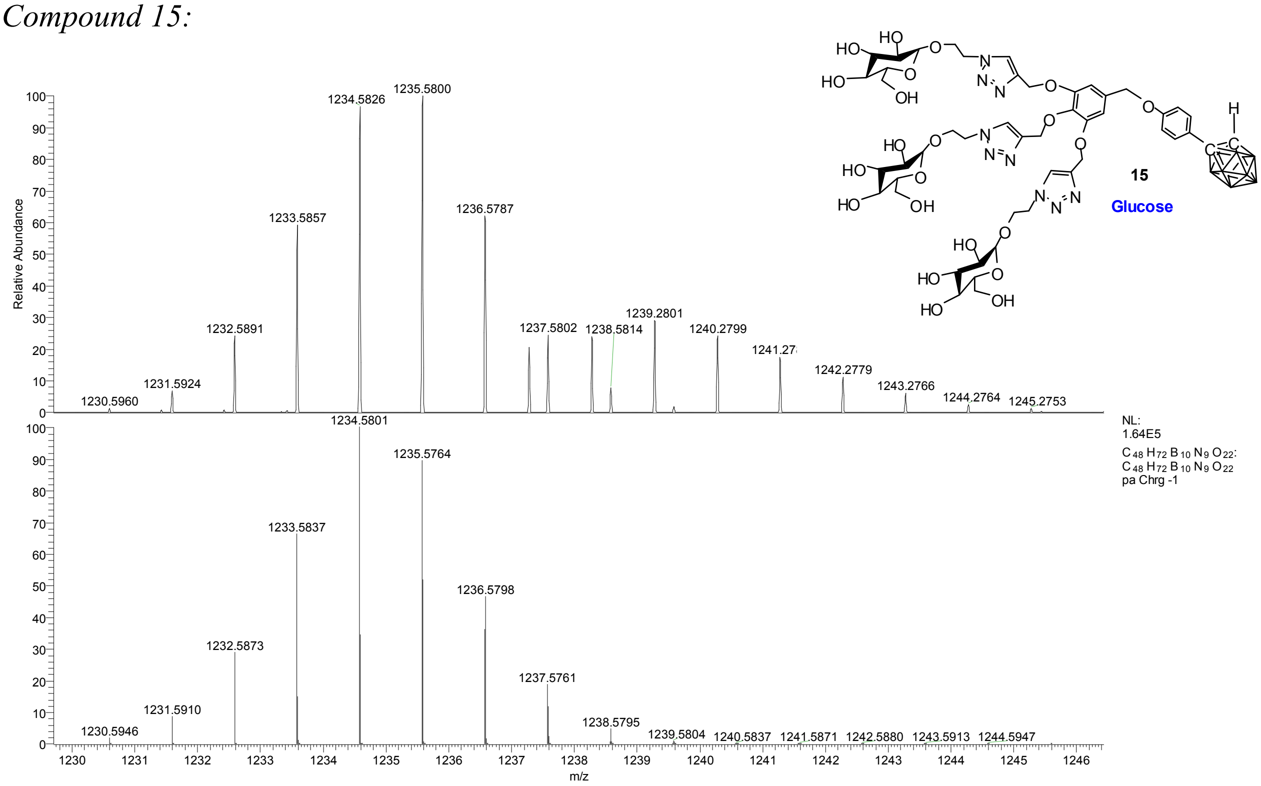

Compound 16:

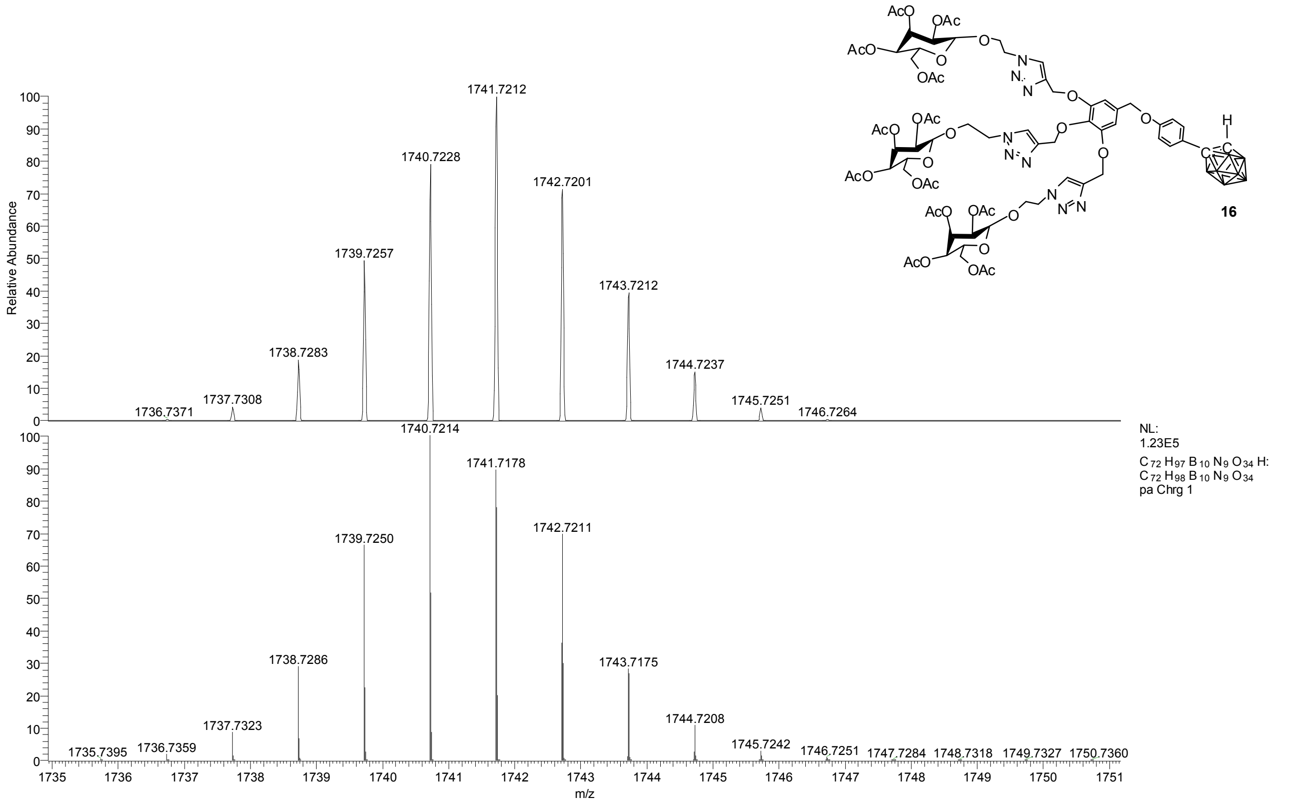

Compound 17:

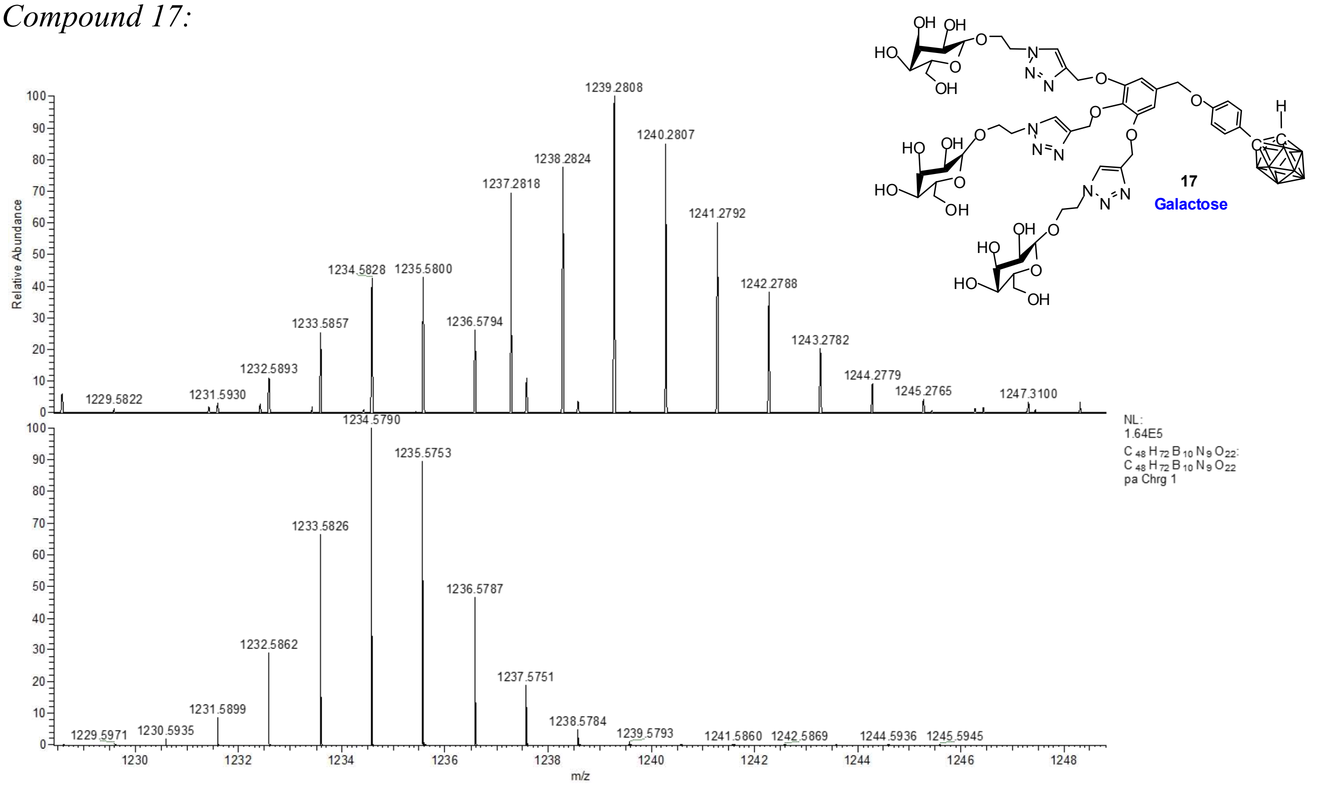

Compound 18:

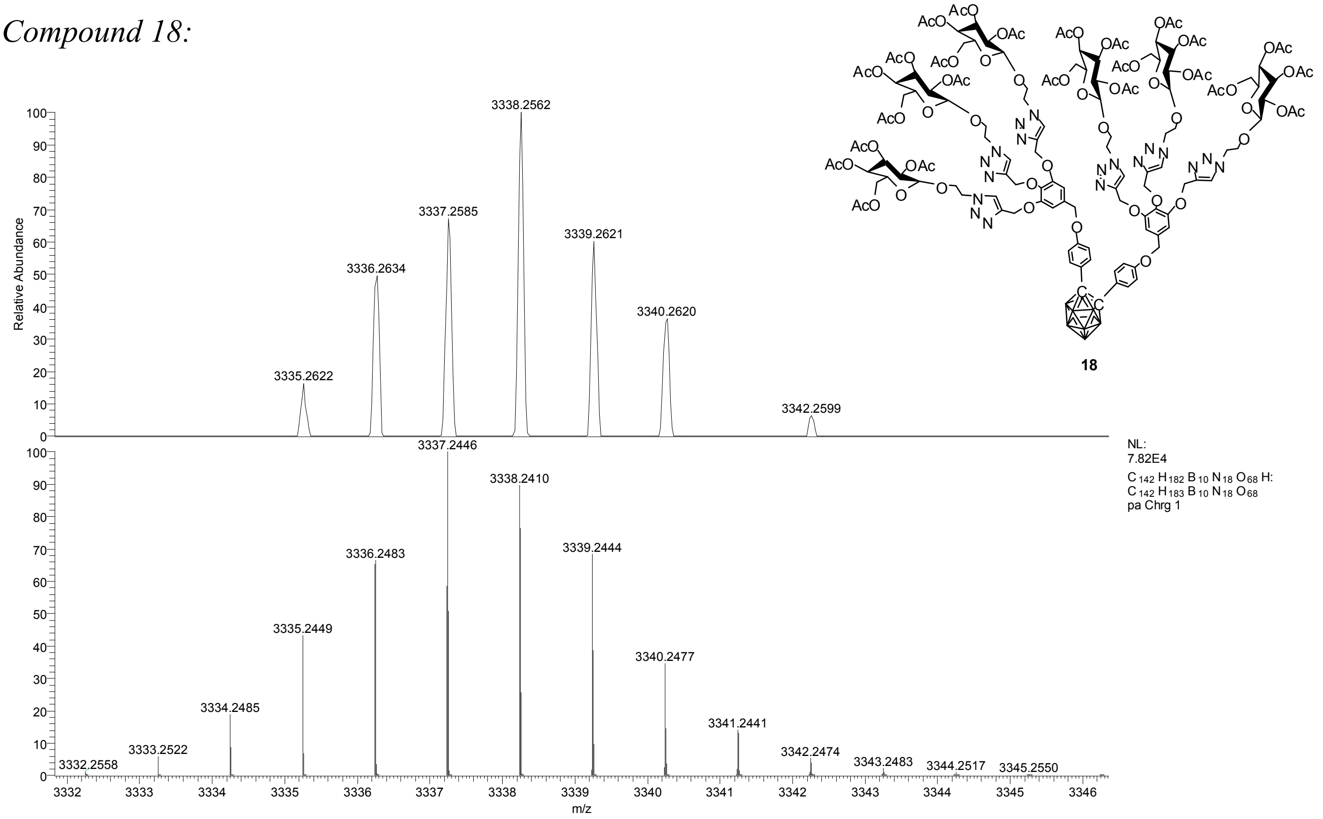

Compound 19:

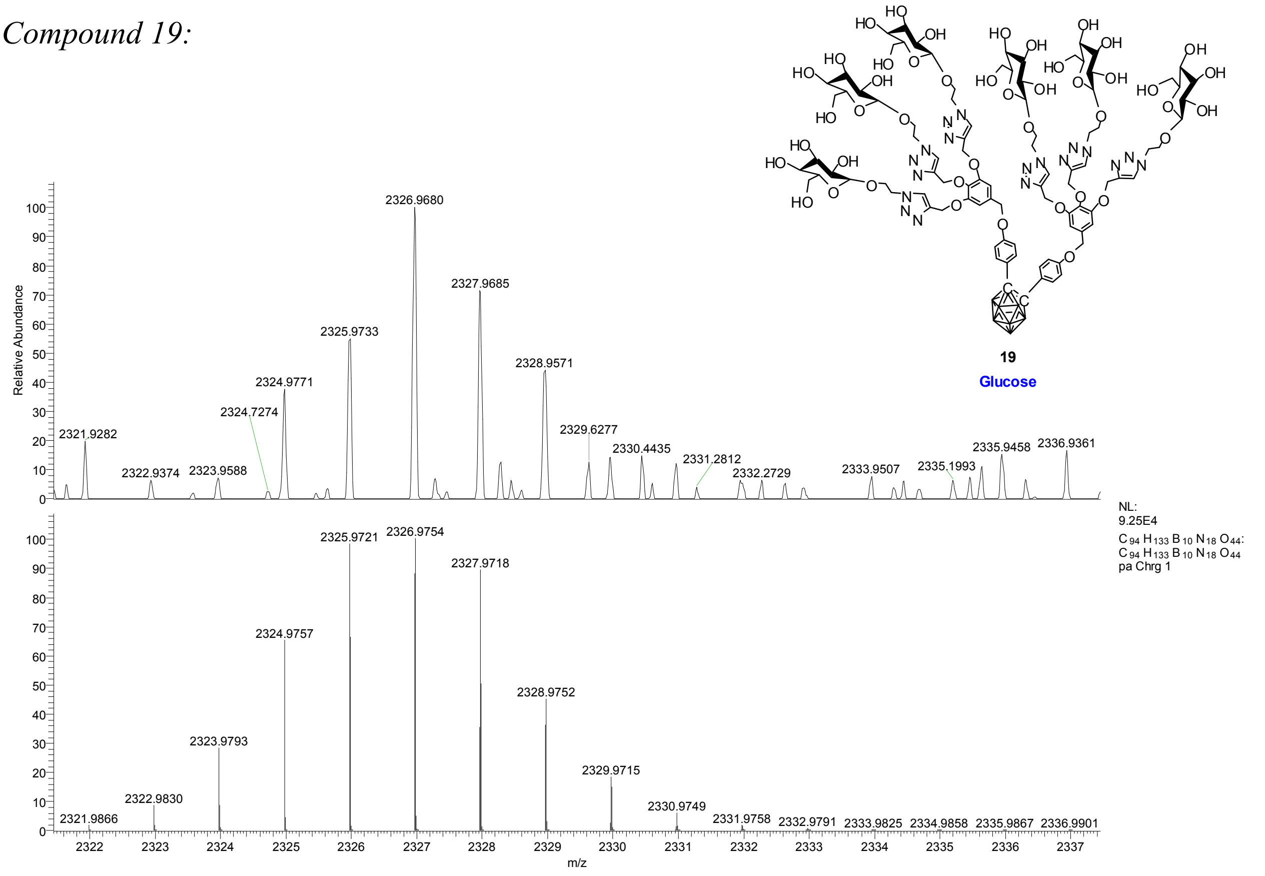

Compound 20:

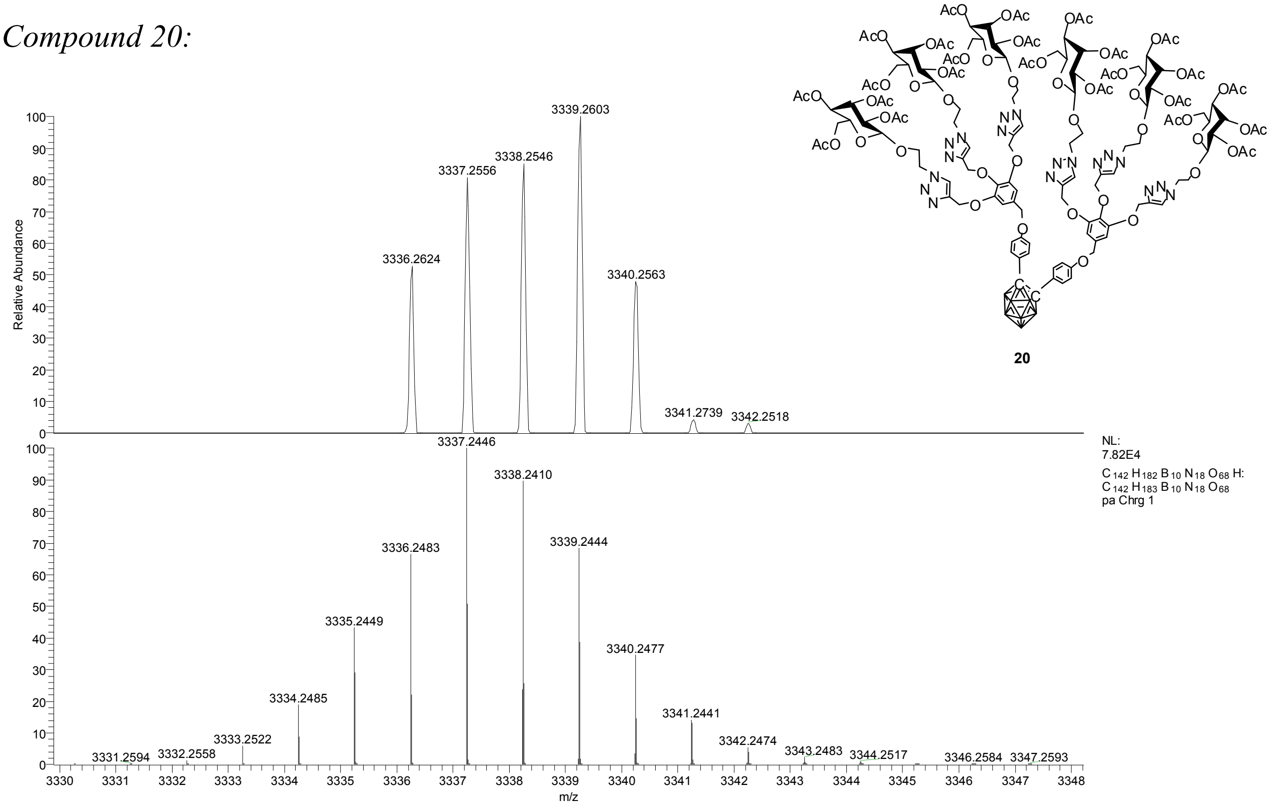

Compound 21:

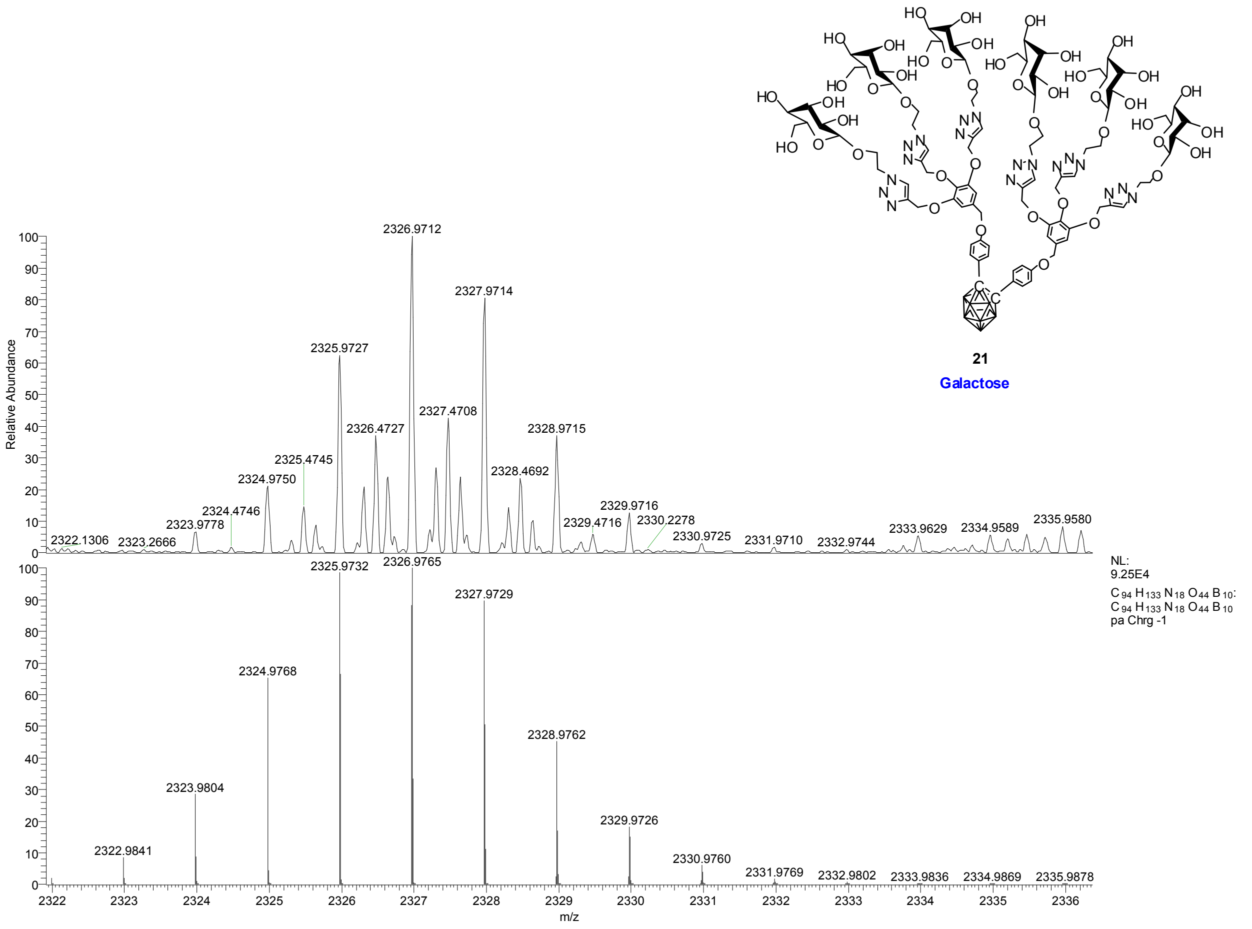
21
Galactose
Relative Abundance
2326.9712
2327.9714
2325.9727
2326.4727
2327.4708
2328.9715
2325.4745
2324.9750
2328.4692
2324.4746
2323.9778
2329.9716
2330.2278
2329.4716
2322.1306
2323.2666
2330.9725
2331.9710
2332.9744
2333.9629
2334.9589
2335.9580
NL:
9.25E4
pa Chrg -1
2325.9732
2326.9765
2327.9729
2324.9768
2328.9762
2323.9804
2329.9726
2322.9841
2330.9760
2331.9769
2332.9802
2333.9836
2334.9869
2335.9878
m/z
